# Clearing the path for whole-mount labeling and quantification of neuron and vessel density in adipose tissue

**DOI:** 10.1242/jcs.263438

**Published:** 2025-02-07

**Authors:** Thomas Rauchenwald, Pia Benedikt-Kühnast, Sandra Eder, Gernot F. Grabner, Sebastian Forstreiter, Michaela Lang, Roko Sango, Tobias Eisenberg, Thomas Rattei, Arvand Haschemi, Heimo Wolinski, Martina Schweiger

**Affiliations:** ^1^ Institute of Molecular Biosciences, University of Graz, 8010 Graz, Austria; ^2^ Institute for Diabetes and Cancer, Helmholtz Center Munich, 85764 Neuherberg, Germany; ^3^ Gottfried Schatz Research Center, Molecular Biology and Biochemistry, Medical University of Graz, 8010 Graz, Austria; ^4^ Centre for Pathobiochemistry and Genetics, Medical University of Vienna, 1090 Vienna, Austria; ^5^ Centre for Microbiology and Environmental Systems’ Science, University of Vienna, 1030 Vienna, Austria; ^6^ Doctoral School in Microbiology and Environmental Science, University of Vienna, 1030 Vienna, Austria; ^7^ Field of Excellence BioHealth - University of Graz, 8010 Graz, Austria; ^8^ BioTechMed-Graz, 8010 Graz, Austria; ^9^ Department of Laboratory Medicine (KILM), Medical University of Vienna, 1090 Vienna, Austria

**Keywords:** Whole-mount imaging, Adipose tissue clearing, Spatial analysis, Image processing, Network density, Quantitative microscopy

## Abstract

White adipose tissue (WAT) comprises a plethora of cell types beyond adipocytes forming a regulatory network that ensures systemic energy homeostasis. Intertissue communication is facilitated by metabolites and signaling molecules that are spread by vasculature and nerves. Previous works have indicated that WAT responds to environmental cues by adapting the abundance of these ‘communication routes’; however, the high intra-tissue heterogeneity questions the informative value of bulk or single-cell analyses and underscores the necessity of whole-mount imaging. The applicability of whole-mount WAT-imaging is currently limited by two factors – (1) methanol-based tissue clearing protocols restrict the usable antibody portfolio to methanol-resistant antibodies and (2) the vast amounts of data resulting from 3D imaging of whole-tissue samples require high computational expertise and advanced equipment. Here, we present a protocol for whole-mount WAT clearing, overcoming the constraints of antibody-methanol sensitivity. Additionally, we introduce TiNeQuant (for ‘tissue network quantifier’) a Fiji tool for automated 3D quantification of neuron or vascular network density, which we have made freely available. Given TiNeQuants versatility beyond WAT, it simplifies future efforts studying neuronal or vascular alterations in numerous pathologies.

## Teaser

**Summary:** A presentation of a whole-mount WAT clearing method that does not require tissue perfusion and expands the usable antibody portfolio. TiNeQuant is presented as an automated tool for 3D quantification of network density.

## INTRODUCTION

White adipose tissue (WAT), the bodies major energy storage depot is comprised primarily of triglyceride (TG)-storing adipocytes. WAT is populated with a diverse array of immune cells, lymphatic and blood vessels, and neurons (AlZaim et al., 2023; Escobedo and Oliver, 2017; Jiang et al., 2017; Massier et al., 2023; Robciuc et al., 2016), whose functions are tightly interconnected with the maintenance of WAT homeostasis. An increase in the mobilization of stored TG to liberate energy substrates involves catecholamines (epinephrine and norepinephrine) that bind to β-adrenergic receptors (Schweiger et al., 2006; Vaughan et al., 1964; Zimmermann et al., 2004). Epinephrine is primarily produced by the adrenal medulla and secreted into the circulation. Alternatively, norepinephrine is released from peripheral sympathetic neurons that spread through the adipose tissue (Frühbeck et al., 2014; Martinez-Sanchez et al., 2022). Besides this well-established regulatory circuit of neuron- or vasculature-delivered catecholamines, the recently identified cellular heterogeneity and immunometabolic signaling axes, suggest a complex interplay between immune cells and adipocytes regulating WAT metabolism (Henriques et al., 2020; Martinez-Sanchez et al., 2022; Massier et al., 2023; Pirzgalska et al., 2017). To study WAT metabolism, bulk protein and mRNA expression analyses, such as western blotting analysis or bulk RNA sequencing are commonly performed. However, these techniques do not allow the identification of cell types accountable for observed metabolic effects. More sophisticated approaches, like flow cytometry or single-cell RNA sequencing, can preserve cellular information, however spatial information is lost during the preparation of cell suspensions (Huesing et al., 2021). Moreover, these methods are not suited for studying neurons, as their cell body is located in sympathetic chain ganglia outside WAT. Although immunohistochemistry delivers spatial information of distinct cell types in their tissue context, neurons and vasculature cannot be adequately captured within the constraints of histological slices, owing to their elongated net-like structure. These limitations underscore the necessity for whole-mount preparation, immunolabeling and clearing to investigate the plasticity of sympathetic neuron and vasculature density in WAT. In recent years, substantial efforts and advancements have been made in the field of tissue clearing and imaging of adipose tissue depots (Chi et al., 2018a; Huesing et al., 2021; Jiang et al., 2017; Willows et al., 2021). Even though sympathetic innervation has been studied for many years, the plasticity of sympathetic neurons in WAT remains a topic of debate. Whereas some studies argue for a substantial adaptability in sympathetic neuron density, thereby regulating the sympathetic stimulus during cold adaptation in WAT (Cao et al., 2018), these findings have not been affirmed by other researchers (Blaszkiewicz et al., 2019; Willows et al., 2021). Notably differences in outcome can be based on antibody sensitivity and specificity, imaging resolution and regional variability of sympathetic innervation across the tissue. To properly address this aspect of WAT biology, the sympathetic neuron or vasculature density in whole-mount cleared WAT can be measured, either manually (Bonda et al., 2020) or, as we show here, in an automated and high-throughput approach. The large amount of acquired data and the susceptibility to (experimenter-based) biased image analysis highlights the need for an accessible, robust and computationally inexpensive open-source pipeline for unbiased 3D quantification of the vessel and neuron network in WAT. Although significant efforts and advances have been undertaken to address this need (Narotamo et al., 2024; Spangenberg et al., 2023), current methods have demonstrated limited robustness and adaptability due to statistics-based segmentation, or are highly computationally expensive, making them difficult to apply to whole-mount datasets. In the field of adipose tissue clearing, Adipo-clear is considered the current gold standard, offering robust performance (Chi et al., 2018a). However, we encountered significant challenges with antibody compatibility, particularly due to the methanol utilized in Adipo-Clear.

Here, we present two adipose tissue clearing protocols that complement Adipo-clear. These protocols offer the advantage of avoiding transcardiac perfusion, a specialized technique that requires training, surgical procedures and is often subject to local animal experimentation laws. Our protocols, adapted from Adipo-Clear, iDISCO and 3DISCO (Chi et al., 2018a; Ertürk et al., 2012; Renier et al., 2014), facilitate broader application and experimental accessibility and are suitable for the use with both methanol-sensitive and methanol-insensitive antibodies. Additionally, we present TiNeQuant (for ‘tissue network quantifier’), a Fiji tool to quantify 3D neuron or vascular density in an unbiased, automated, robust and user-friendly fashion, which is freely available at https://github.com/SchweigerLab/TiNeQuant . In our pipeline, we deploy LABKIT (Arzt et al., 2022), an easy-to-use supervised learning segmentation plugin featuring graphics processing unit (GPU) acceleration via CLIJ (Haase et al., 2020). This allows the user to train segmentation classifiers on their own datasets, allowing robust image segmentation across diverse input datasets. We anticipate that the integration of our protocols and this new bioinformatics tool will advance the exploration of neuronal and vascular heterogeneity in adipose tissue and improve statistical power of future quantitative analyses.

## RESULTS

### Tissue clearing methods suiting immunolabeling with methanol-resistant and methanol-sensitive antibodies

**Fig. 1.**
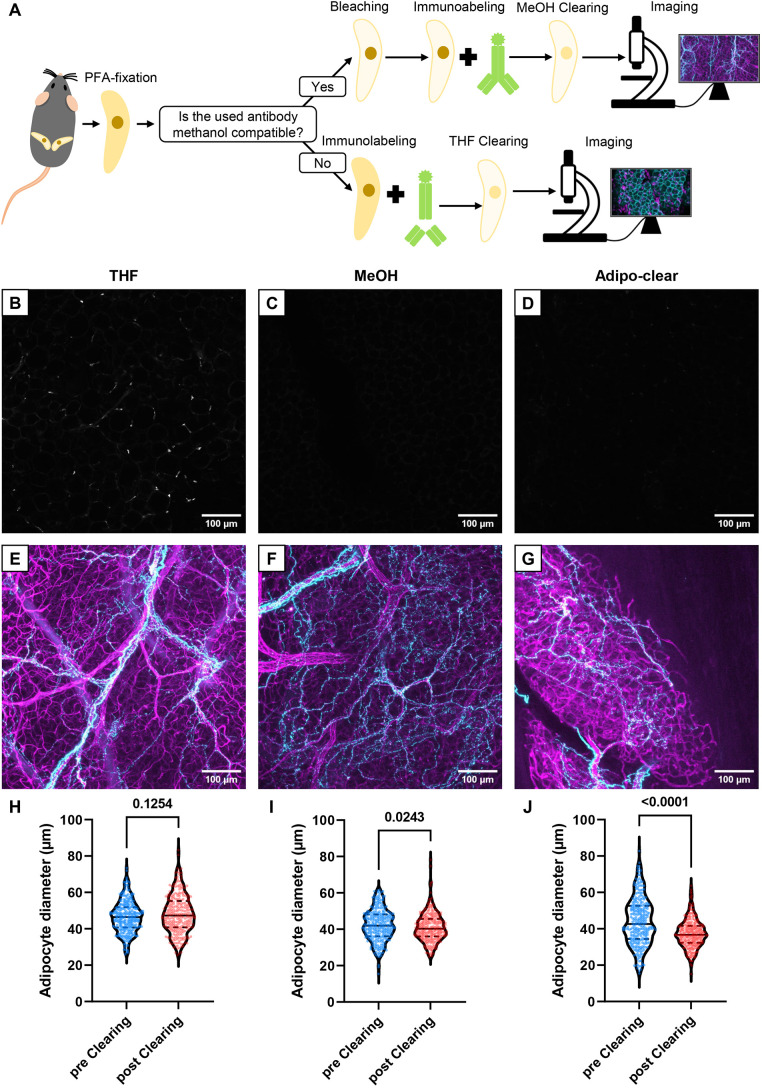
**Tissue clearing protocols suitable for methanol-sensitive and methanol-insensitive antibodies.** (A) Flow chart illustrating our two methods for immunolabeling and clearing WAT depending on whether the antibody used is methanol sensitive or methanol resistant. (B–D) Representative iWAT depots stained with the methanol-sensitive anti-CD68 antibody processed with the THF protocol for methanol-sensitive antibodies (B), the MeOH-protocol for methanol-resistant antibodies (C) or Adipo-clear (D). Images were recorded using identical settings and imaging depth. (E–G) Representative iWAT depots stained with anti-tyrosine hydroxylase (cyan) and anti-CD31 (magenta) antibodies, processed with the THF protocol (E), the MeOH protocol (F) or Adipo-clear (G). (H–J) Violin plots with median (solid line) and quartiles (dashed lines) showing adipocyte diameter before and after clearing with the THF protocol (H), the MeOH protocol (I) or Adipo-clear (J). 300 adipocytes across five randomly selected regions in three biological replicates per group were measured. A Shapiro–Wilk test was performed to test for normal distribution, then a Mann–Whitney *U*-test was performed to analyze the non-normally distributed data.

The commonly used methanol-based tissue clearing protocols limit the antibody portfolio for tissue labeling to methanol-insensitive antibodies. In this study, we assessed various clearing approaches applicable to murine WAT. We employed a method based on Adipo-clear (Chi et al., 2018a) and iDISCO (Renier et al., 2014) to image samples using antibodies that are methanol resistant and a method based on 3DISCO (Ertürk et al., 2012) tissue clearing to image antibodies that are sensitive to methanol and compared the results with the established Adipo-clear protocol. The workflow for both variants of the protocol is illustrated in Fig. 1A and thoroughly described in the Materials and Methods section.

Similar to what has previously been described for other tissues (Renier et al., 2014), we observed that a bleaching step after methanol-mediated dehydration considerably lowered autofluorescence in WAT (Fig. S1A). Incorporating the bleaching step into the methanol-free protocol, however, was not feasible owing to the potential formation of highly explosive organic peroxides by the reaction of tetrahydrofuran (THF) with hydrogen peroxide. As anticipated, the methanol-sensitive antibody CD68 was only detected in WAT that was cleared using our THF protocol and was not detected upon using methanol-based protocols (our MeOH protocol and Adipo-clear) (Fig. 1B–D). We did not find differences regarding quality of labeling (Fig. 1E–G) or clarity (Fig. S1B) between the THF, MeOH and Adipo-clear protocols using the methanol insensitive CD31 and tyrosine hydroxylase (TH) antibodies. To test whether the clearing process affects WAT morphology, we measured adipocyte diameters of WAT pre and post clearing. All protocols applied increased the stiffness of WAT (data not shown). Moreover, we found significant adipocyte shrinkage using our MeOH (−2.8%) and the Adipo-clear protocol (−15.0%) but no difference in adipocyte size using our THF protocol (Fig. 1H–J). Together, our newly established workflows for WAT clearing perform without the need for transcardiac perfusion and largely preserve adipocyte morphology, while providing comparable tissue clarity and quality of labeling to gold-standard protocols. Furthermore, our THF-based WAT clearing protocol addresses methanol sensitivity issues, thus expanding the range of antibodies that can be applied for immunolabeling in combination with WAT clearing.

### A new tool for the quantification of neuron and vasculature density

**Fig. 2.**
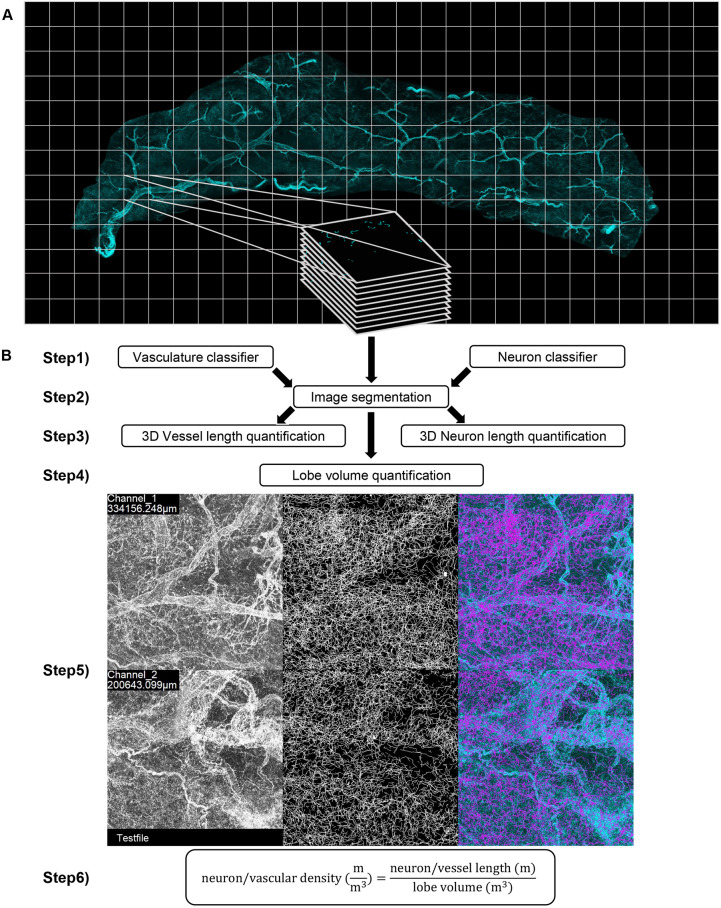
**Workflow overview for the quantification of neuron and vasculature density.** (A) Recording of image stacks. (B) Step 1. Training of classifiers for structures intended to be quantified. Step 2. Segmentation of images using random forest-based image segmentation. Step 3. Quantification of neuron and vessel length. Step 4. Quantification of the depicted lobe volume. Step 5. Optical evaluation of the quantification results using merged images of maximum intensity projections of the input data (left), the calculated skeleton (middle) and the overlay of the two (right). Step 6. Calculation of neuron or vascular density per lobe volume.

Two-dimensional (2D) quantification of network density of WAT is not sufficient, owing to signal overlap (Fig. S2A,B). However, the large amount of data generated by 3D imaging of whole-tissue samples is challenging for manual annotation and quantification. Accordingly, there is a call for a robust, automated and computationally inexpensive tool to quantify neuron or vascular density from 3D images. We established an image processing pipeline to quantify the density of sympathetic neurons and vasculature. In Fig. 2, the workflow for the quantification of two-channel image stacks depicting both blood vessels (CD31-positive cells) and sympathetic neurons (TH-positive cells) is shown. We also provide a version of this pipeline for cases where only single-channel image data is quantified. In the following example, the six-step process of TiNeQuant is applied as visualized in Fig. 2B. In step 1, the user trains classifiers on their own training data by marking foreground and background areas in LABKIT followed by training and saving the classifier with the desired classification settings. (For more information on how to train a classifier in LABKIT and best practice examples, visit https://imagej.net/plugins/labkit/.) In step 2, the classifier is employed within the pipeline for random forest-based image segmentation. In step 3, based on the segmented image stacks, the pipeline calculates the length of neurons and blood vessels in 3D space. Step 4 uses the combined segmentation results of both the vasculature and the neuron channel to calculate the lobe volume of the image stack (Fig. S3C). For step 5, the quantification result is evaluated optically using a merged image displaying a maximum intensity projection of the input data, the calculated skeleton and the overlay of the two. In step 6, the output data is used to calculate the neuron and/or vascular density per lobe volume. The results, as well as the montages for optical evaluation, as shown in Fig. 2B, are located in the specified output folder.

**Fig. 3.**
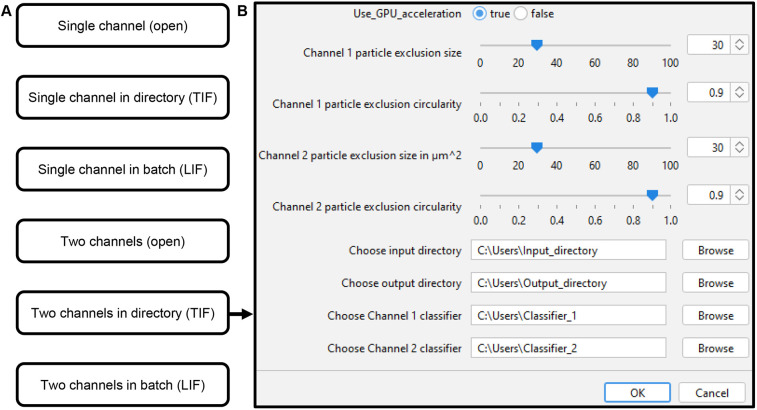
**Data analysis pipeline using various input data formats.** (A) The appropriate program version is chosen depending on the type of input data. (B) Representation of the dialog box. GPU usage, settings for image preprocessing, and the choice of input and output directory as well as the self-trained classifiers can be selected.

By utilizing LABKIT for supervised learning-based image segmentation, this pipeline is broadly applicable and can perform robust image segmentation on diverse input datasets. An overview of the input data options is illustrated in Fig. 3A. After choosing the appropriate TiNeQuant script for the input data (Fig. 3A), it is executed in the Fiji script editor. We included an optional preprocessing step into the pipeline for particle exclusion. In this process, the user defines the upper size limit as well as the circularity of particles that should be excluded from the calculation. The dialog box depicted in Fig. 3B allows the selection of preprocessing options, and to input a choice for the input and output directory as well as the LABKIT segmentation classifiers. The default settings for particle exclusion demonstrated the optimal results in our datasets. In the output directory, folders containing montages for optical evaluation of the skeletonization result, segmented neuron stacks and segmented vasculature stacks are generated. The quantification results for neuron length, vessel length as well as the calculated lobe volume of the image stacks are saved as a .csv file in the output directory.

### Testing and evaluation of TiNeQuant

**Fig. 4.**
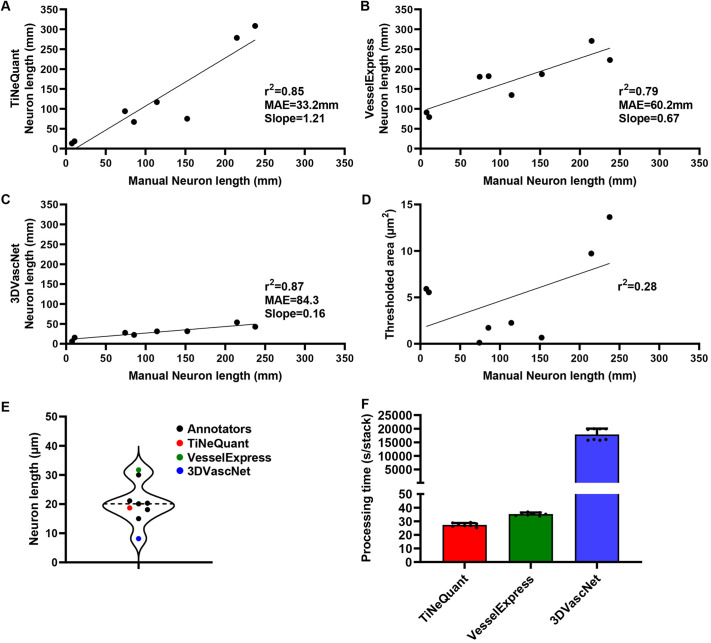
**Evaluation of automated neuron length quantification.** iWAT depots excised from mice housed at 30°C or 4°C for 7 days were processed with our methanol containing (MeOH) clearing protocol. Image stacks of anti-tyrosine hydroxylase antibody labeled neurons were selected evenly between the two housing conditions and randomly selected across the whole depot of three biological replicates. (A–C) Linear regression analysis of manual neuron length quantification plotted against automated neuron length quantification by TiNeQuant (A), Vessel Express (B) and 3DVascNet (C). (D) Linear regression analysis of manual neuron length quantification plotted against the thresholded area (8-bit gray value>170). (E) Accuracy was tested by comparing the results obtained from six independent manual annotators with the results of the automated quantification by our pipeline. A violin plot with median highlighted is shown. (F) Processing time per image stack of TiNeQuant, VesselExpress and 3D VascNet [test dataset contained 8 (*n*=8) image stacks with a total of 1124 512×512 pixels slices]. Graph shows mean+s.d.

To address whether neuron quantification using TiNeQuant is superior to potentially applicable alternative methods, we compared the accuracy to ground truth, processing time and maximum dataset size of TiNeQuant with two recently published, methods, VesselExpress (Spangenberg et al., 2023) and 3DVascNet (Narotamo et al., 2024). Additionally, as a simple and computationally inexpensive possible alternative, we tested the correlation between neuron length and the area of pixels above a fixed threshold (8-bit gray value>170). We randomly selected ten image stacks from two independent inguinal WAT (iWAT) whole-mount scans. Each image stack, measuring 620×620 µm, comprised a sequence of 133 or 148 images, respectively. These image stacks were divided into eight validation and two training stacks. We manually labeled the validation stacks using the pencil tool and calculated the 3D neuron length using the skeletonize3D function in Fiji software. Then we employed TiNeQuant, VesselExpress and 3DVascNet to the identical image stacks. Whereas the segmentation step in VesselExpress mainly relies on statistics-based thresholding and 3DVascNet uses a pretrained classifier, TiNeQuant employed a classifier trained on the two training stacks. We performed linear regression analysis on the outcome by plotting manually measured neuron lengths against the automatically calculated neuron lengths for each of the stacks. This comparison yielded a good level of precision for TiNeQuant (r^2^=0.8531) (Fig. 4A), VesselExpress (r^2^=0.7859) (Fig. 4B) and 3DVascNet (r^2^=0.8720) (Fig. 4C). The thresholded area turned out to be a poor predictor of neuron length (r^2^=0.28) (Fig. 4D). The level of accuracy was much improved in TiNeQuant (mean absolute error=33.2 mm) in comparison with VesselExpress (mean absolute error=60.2 mm) and 3DVascNet (mean absolute error=84.3 mm), with evidence of bias in case of VesselExpress, as indicated by the trendline not intersecting the origin. To further evaluate the accuracy of the automated pipelines, six independent annotators from three different labs performed manual quantification of a randomly selected image stack with a sequence of 16 images measuring 620×620 µm in size. The results of the manual quantification were similar to TiNeQuant (absolute error to mean=2.1 mm), whereas VesselExpress (absolute error to mean=11.0 mm) and 3DVascNet (absolute error to mean=12.6 mm) demonstrated decreased accuracy (Fig. 4E). Upon comparing the processing time for analyzing the test dataset used in Fig. 4A–D, we found that TiNeQuant is the fastest method, requiring an average of 27 s per image stack whereas VesselExpress was 29% slower (35 s per image stack) and 3DVascNet based analysis took 654 times longer (17,888 s per image stack) (Fig. 4F). Accordingly, TiNeQuant excels in terms of accuracy and processing time.

### Analyzing neuronal and vascular density in WAT of mice housed at different ambient temperatures

**Fig. 5.**
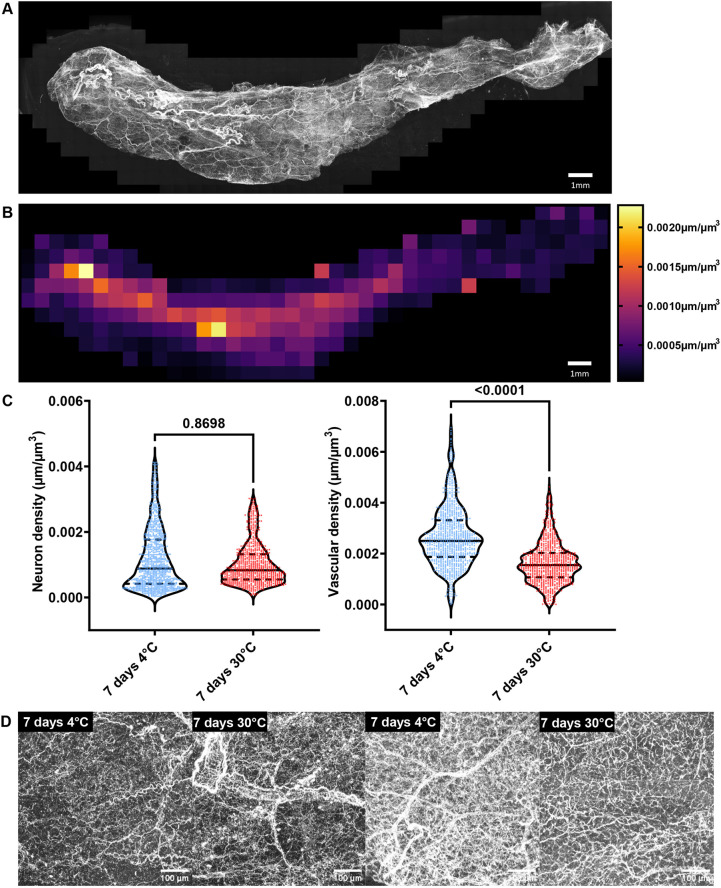
**Analyzing sympathetic innervation and vascular density in iWAT of mice housed at different ambient temperatures.** C57BL/6J mice were either housed at 30°C or at 4°C for 7 days. Mice were killed, and iWAT excised, treated according to the protocol for methanol-insensitive antibodies and immunolabeled using anti-tyrosine hydroxylase and anti-CD31 antibody. (A) Example maximum intensity projection of an anti-tyrosine hydroxylase immunolabeled and whole-mount cleared iWAT sample of a mouse housed at 4°C. (B) Heatmap showing measured neuron density (neuron length per regional lobe volume) of the same sample. (C) Quantification of iWAT neuron and vascular density across ∼850 regions and three biological replicates per group. A Shapiro–Wilk test was performed to test for normal distribution, then a Mann–Whitney *U*-test was performed to analyze the non-normally distributed data. Violin plots display the median (solid line) and quartiles (dashed lines). (D) Representative maximum intensity projections of single tiles that were used for quantification. The chosen examples match the mean neuron and vascular density (from left to right, neuron density at 7 days 4°C mean: 0.00117 m/m^3^ example: 0.00116 m/m^3^; 7 days 30°C mean: 0.00102 m/m^3^ example: 0.00102 m/m^3^, right: vascular density 7 days 4°C mean: 0.00265 m/m^3^ example: 0.00268 m/m^3^, 7 days 30°C mean: 0.00164 m/m3 example: 0.00165 m/m^3^).

It is still a matter of debate whether or not metabolic changes due to differences in ambient temperature are mediated by varying neuronal density in WAT (Blaszkiewicz et al., 2019; Cao et al., 2018; Willows et al., 2021). We addressed this question by using our novel pipeline, and compared neuron and vascular network density in WAT of mice that were housed at thermoneutral (30°C) or cold conditions (4°C) for 7 days. We killed mice, excised iWAT depots and performed whole-tissue imaging of anti-TH- and anti-CD31-stained iWAT according to our protocol (Fig. 1A) for methanol-resistant antibodies (Fig. 5A shows a representative 4°C depot). Using our established pipeline for image processing and quantification, we created heatmaps depicting sympathetic neuron density in its spatial context (Fig. 5B shows a representative 4°C depot). We further used TiNeQuant to analyze neuron and vascular density of the whole iWAT depots and compared cold exposure to thermoneutral housing conditions. We found no significant difference in sympathetic neuron density (*P*=0.8698) but a 36% increase in vascular density (*P*<0.0001) in mice housed in cold compared to mice housed in thermoneutral conditions (Fig. 5C,D). These results demonstrate the applicability of our new method in addressing a biological question.

All together, we present a novel approach that allows the visualization and analysis of the 3D architecture of adipose tissue using methanol-resistant and methanol-sensitive antibodies. With TiNeQuant, we present a robust and user-friendly open-source software tool for the quantification of 3D networks.

## DISCUSSION

The current efforts and accomplishments in spatial transcriptomics have revealed a distinct adipose tissue microarchitecture and demonstrated a spatially dependent interplay between different cell types residing in the tissue (Bäckdahl et al., 2021). However, the restriction to two dimensions in spatial transcriptomics and immunohistochemistry limit morphologic analyses and the visualization and quantification of elongated structures such as blood vessels, lymphatic vessels and neurons. As sympathetic innervation and vascularization of WAT is crucial for metabolic health, studying the 3D network of these cell types is of high interest for the scientific field but currently restricted to image analysis experts.

The adipose tissue clearing approach presented here allows the visualization and analysis of the 3D architecture of adipose tissue using methanol-resistant and methanol-sensitive antibodies without the need for transcardiac perfusion. This enables the exploration of the spatial localization and interaction of different cell types within adipose tissue. Additionally, we developed TiNeQuant, a robust and user-friendly software tool for the quantification of 3D networks, based on the open-source platform Fiji.

We tested our adipose tissue clearing approach in comparison to the current gold standard, Adipo-clear. Whereas methanol-based protocols, including Adipo-clear proved ineffective with methanol-sensitive antibodies, we observed no discernible difference in terms of overall clarity of the tissue and labeling quality using methanol-insensitive antibodies. Importantly, our adipose tissue clearing approach minimized adipocyte shrinkage. Although transcardiac perfusion appears to be essential for clearing blood- and muscle-rich tissues to remove chromophores like heme (Richardson et al., 2021), we found that this step is not generally necessary for adipose tissue. However, certain antibodies might interfere with erythrocytes or erythrocyte remnants and cause unspecific fluorescence signal. In these very specific cases, transcardiac perfusion might be warranted.

Previous automated approaches for 2D quantification (Willows et al., 2021), and the current standard for 3D network quantification, VesselExpress (Spangenberg et al., 2023), demonstrated unbiased results, yet their applicability across different datasets is hampered by the constraints of histogram-based thresholding for image segmentation. Furthermore, quantifying 2D data is error prone as projection methods inherently cause information loss, leading to underestimating the density of the tissue network. Commercial software systems such as Amira (Thermo Fisher Scientific), Aivia (Leica Microsystems), Imaris (Oxford Instruments) or Arivis Pro (ZEISS) are potentially capable of processing and quantifying large-scale 3D neuronal data sets including the application of machine learning approaches for image segmentation, yet their purchase entails significant costs and they are typically less flexible regarding the implementation of custom features than open-source software systems. Previous studies have used FilamentTracer, a component of the commercial software package Imaris for the quantification of sympathetic nerve density (Chi et al., 2018b; Xie et al., 2022). However, these tracing tools are used on limited randomly sampled cubes, presumably due to inapplicability of filament tracing algorithms on whole-mount samples, which would necessitate unsurmountable computational requirements. Alternative approaches, such as Python-based tools (Jaeschke et al., 2022), hold high potential for future implementation of potent deep-learning libraries for image segmentation including TensorFlow (Abadi et al., 2016 preprint) or PyTorch (Paszke et al., 2019 preprint). However, current versions only implement manual gray-value thresholding or unsupervised histogram-based image segmentation methods, and the construction as a command-line tool also results in a high entry barrier for coding-inexperienced users. One limitation of deep learning approaches is their reliance on extensive manually labeled ground-truth datasets for training. 3DVascNet (Narotamo et al., 2024), a recent and promising method, utilizes the 3D CycleGAN model, which only requires 2D ground-truth masks for training. Tests using their pretrained model were promising; however, even on high-performance workstations including GPU usage, this pipeline remained computationally expensive, making its application for whole-mount segmentation impractical at this stage. Upon comparing our TiNeQuant pipeline with 3DVascNet and VesselExpress, we found TiNeQuant to be the most accurate and the fastest option. The estimated calculation time for processing an average whole-mount scanned adipose tissue depot using 3DVascNet is 1740 h 6 min, whereas VesselExpress takes 3 h 26 min and TiNeQuant completes the same operation in 2 h 40 min. One further consideration is the maximum processable dataset size, which is most limited by RAM usage. For a test stack of 37 images measuring 512×512 pixels, the peak RAM usage was 3 GB using TiNeQuant, 5 GB for VesselExpress and 95 GB for 3DVascNet. The supervised learning-based image segmentation of TiNeQuant offers 3D segmentation that can easily be tailored to the input data from the user. A limitation of the shallow learning approach used by TiNeQuant is that too much training data often leads to deteriorating segmentation results, which can be managed following the best practice examples in the LABKIT documentation (Arzt et al., 2022). Although deep learning-based image segmentation could potentially lead to increased segmentation quality, the minimal training data and limited computational resources needed for TiNeQuant drastically increase usability irrespective of the availability of image analysis experts or high-end workstation computers. We used our clearing pipeline and TiNeQuant to compare neuron and vasculature density in WAT of cold-exposed mice and mice kept at thermoneutral conditions for 7 days. In WAT, sympathetic nerve activation induces catabolic remodeling characterized by increased lipolysis and recruitment of ‘thermogenic’ adipocytes (Willows et al., 2023), whereas blood vessels enable endocrine communication, substrate supply and prevent hypoxia of WAT. Previous studies have suggested that enhanced intraadipose sympathetic activity is due to axon outgrowth in cold-exposed and cachectic animals (Cao et al., 2018; Xie et al., 2022). In these approaches, random WAT regions were selected for quantification. Owing to the heterogeneous distribution of sympathetic neurons in adipose tissue, sole quantification of a randomly selected subfraction of the tissue might not be sufficient for reliable comparative analyses. In contrast, our approach setting neuron length in relation to local lobe volume provides information about spatial heterogeneity and allows robust global density quantification in the whole-tissue sample in an automated and unbiased manner. When performing whole-mount analysis, we did not observe a global increase in sympathetic neuron density in WAT upon cold exposure, which is consistent with recent findings (Blaszkiewicz et al., 2019; Willows et al., 2021). It must be noted that Blaszkiewicz et al. and Willows et al. used the pan-neuronal markers PGP9.5 and β-3 tubulin, whereas Cao et al. and Xie et al. used pan-neuronal (synaptophysin), mostly sympathetic neuron-specific (TH), markers as well as stathmin-2 and growth-associated protein-43 as markers for axon outgrowth. In our hands, of all the antibodies tested, TH was the most sensitive option for staining sympathetic neurons. This is crucial, as axonal outgrowth predominantly affects only the smallest neurons. One major caveat is that TH levels rise during cold exposure, so highly sensitive imaging is essential to avoid confusion from increased neuron detectability caused by higher protein expression. The fact that we did not detect increased neuron density in cold-exposed mice indicates that we achieved a sensitivity level sufficient to detect the smallest axons, which are those most likely to change during metabolic adaptation. In comparison to light-sheet microscopy (Cao et al., 2018; Xie et al., 2022), the use of confocal laser scanning microscopy, as employed in our study and by others (Blaszkiewicz et al., 2019; Willows et al., 2021), allows higher sensitivity and resolution and leads to the capture of finer structures. However, it is possible that even more sensitive approaches might challenge our current views in the future.

Blood vessels regulate the transport of nutrients, oxygen, growth factors, cytokines and hormones that are required for proper WAT function. Additionally, they transport adipokines and fatty acids from WAT to other organs affecting whole-body energy homeostasis (Corvera et al., 2022). We are the first to demonstrate significantly higher 3D vascular density in WAT of cold-exposed mice compared to mice kept at thermoneutral conditions. However, this finding is in agreement with those from previous studies which have described increased vessel area in immunohistochemically stained WAT slides after 1 week of cold exposure (Luo et al., 2017; Xue et al., 2009). These studies concluded that cold-induced angiogenesis depends on sympathetic activation and is crucial for non-shivering thermogenesis in mice. We are confident that our tissue clearing protocol in combination with TiNeQuant will advance future studies on vascularization and innervation, and their role in maintaining WAT homeostasis under physiological and pathophysiological conditions.

This work enables the analysis of spatial heterogeneity of a plethora of different cell types and to quantify blood vessel, lymphatic vessel or neuron length in 3D datasets, accelerating the application of tissue clearing for the holistic examination of organs across a wide range of research disciplines.

## MATERIALS AND METHODS

### Animals and tissue preparation

Male C57Bl/6J mice [Janvier Laboratories, CSAL (Orleans) - 1993 (F172)] of 10 to 11 weeks of age were housed at 22°C or, if indicated, at thermoneutral conditions (30°C) or exposed to cold (4°C) for a period of 7 days. We randomly allocated samples or animals to experimental groups, ensuring an equal average body weight across groups. Mice subjected to cold were housed singularly to ensure consistent exposure to the environment. Experiments using cold-exposed mice were reproduced in two independent experiments. Mice were killed by cervical dislocation; the inguinal WAT was dissected and drop-fixed for 24 h in 1% paraformaldehyde (PFA) in phosphate-buffered saline (PBS) at 4°C without shaking to preserve tissue shape. Fixed tissue was stored in PBS supplemented with 0.05% sodium azide. Animal study protocols were approved by the Austrian Federal Ministry for Science, Research, and Economy (protocol numbers BMBWF-66.007/0005-V/3b/2019) and were conducted in compliance with the council of Europe Convention (ETS 123).

### Sample pretreatment for immunolabeling and clearing

After excision and PFA fixation, tissues were washed three times for 1 h in PBS supplemented with 10 U/ml heparin at gentle shaking.

#### Pretreatment for sample clearing with methanol

Samples were dehydrated using a methanol ladder, starting with 20% methanol diluted in double-distilled (dd)H_2_O, for 30 min at room temperature (RT) with gentle shaking. Thereafter, samples were subjected to 40% methanol for 30 min, followed by 60% methanol for 30 min, 80% methanol for 30 min, and finally twice in 100% methanol for 30 min each time. Next, to effectively reduce autofluorescence, the samples were bleached using 5% hydrogen peroxide in 100% methanol supplemented with 10 mM EDTA at pH 8 for 48 h at 4°C. Samples were rehydrated with methanol diluted in ddH2O by gentle shaking, starting with 80% methanol for 30 min at RT, followed by 60% methanol, 40% methanol and 20% methanol. Samples were washed twice for 1 h in PBS supplemented with 0.2% Triton X-100. Finally, samples were permeabilized in PBS containing 0.2% Triton X-100, 20% DMSO and 300 mM glycine at 37°C on a rotating wheel for 24 h.

#### Pretreatment for sample clearing without methanol

Samples were permeabilized in PBS containing 0.2% Triton X-100, 20% DMSO and 300 mM glycine for 24 h on a rotating wheel at 37°C. Owing to the risk of forming highly explosive and shock-sensitive organic peroxides, bleaching of the samples using hydrogen peroxide is not recommended upon tetrahydrofuran-based dehydration.

### Immunolabeling

To avoid unspecific antibody binding, tissues were blocked in a solution of 0.2% Triton X-100 in PBS containing 5% horse serum (Thermo Fisher Scientific, #16050122) for 24 h at 37°C on a rotating wheel. Next, the tissues were incubated with the primary anti-CD68 antibody (1:500, Bio-Rad, #MCA1957GA), or anti-tyrosine hydroxylase (1:1250, Sigma-Aldrich, #AB-152) and anti-CD31 (1:300, Biotechne, #AF3628) antibodies in PBS containing 0.2% Tween 20, 1 U/ml heparin, 5% DMSO and 5% horse serum for 72 h at 37°C on a rotating wheel. Thereafter, samples were washed five times for 2 h each at RT in PBS containing 0.2% Tween 20 and 1 U/ml heparin. Tissues were subsequently incubated with the secondary goat anti-rabbit-IgG Alexa Fluor 647 (1:500, Thermo Fisher Scientific, #A-21246) and goat anti-rat-IgG Alexa Fluor 488 (1:500, Thermo Fisher Scientific, #A-48262) or donkey anti-goat-IgG Alexa Fluor 488 (1:500, Thermo Fisher Scientific, #A-11055) antibodies in PBS supplemented with 0.2% Tween 20, 1 U/ml heparin and 5% horse serum for 72 h at 37°C on a rotating wheel. Samples were again washed five times for 2 h each in PBS containing 0.2% Tween 20 and 1 U/ml heparin, followed by an overnight wash in the same solution. Finally, the samples were embedded with 1% agarose prepared in PBS.

### Sample clearing

#### Clearing with methanol

The dehydration process was performed at RT under gentle shaking by incubating the embedded samples with 20% methanol in ddH_2_O for 2 h, followed by 40% methanol for 2 h, 60% methanol for 2 h, 80% methanol for 2 h and twice with 100% methanol for 1 h each. Subsequently, the samples were incubated in 50% dichloromethane in methanol for 2 h, and then twice for 30 min in 100% dichloromethane. Finally, the samples were incubated for at least 12 h in 100% dibenzylether under slight shaking at 4°C. The samples remained stable for imaging for at least 5 days when stored at 4°C.

#### Clearing without methanol

Embedded samples were dehydrated at RT using tetrahydrofuran (Sigma-Aldrich, 186562-1L) with gentle shaking. First, the samples were incubated with 50% tetrahydrofuran in ddH_2_O for 2 h, followed by 75% tetrahydrofuran for 2 h, and finally with 100% tetrahydrofuran twice for 1 h each time. A gentler gradient with shorter incubation times might preserve the tissue morphology even better. Thereafter, samples were incubated in 100% dichloromethane for 1 h and then twice for 30 min each. Finally, samples were incubated in 100% dibenzylether under slight shaking at 4°C for at least 12 h. The samples can be stored at 4°C and remain stable for imaging for at least 14 days without signal deterioration (Fig. S3).

### Adipo-Clear

Following the Adipo-Clear protocol, mice were deeply anesthetized with ketamine-xylazine and transcardiac perfusion was performed with ∼20 ml PBS until the blood was removed from the tissue. Then the perfusate was switched to 4% PFA in PBS and perfusion was stopped when the mouse significantly stiffened. iWAT depots were dissected and post-fixed in 4% PFA in PBS overnight at 4°C. The Adipo-Clear protocol was then followed for small tissue as described by Lin et al. (2022), using identical antibodies and concentrations to those specified above.

### Image acquisition

Confocal images were captured on a Leica DMi8 DLS (Leica Microsystems, Wetzlar, Germany) confocal laser scanning microscope. Fluorescence labels were excited with an argon laser (488 nm) and a helium neon laser (633 nm), respectively. Emission spectra was set to ensure minimum crosstalk (505–535 nm for Alexa Fluor 488, 660–720 nm for Alexa Fluor 647). A decrease in signal intensity along the *z*-axis of the image stack was compensated by a preset linear adjustment of the detector gain enabling even exposure across the whole-tissue depth. Whole iWAT samples were scanned using photomultiplier tube-detectors, using a HC FLUOTAR L 25x objective (NA 0.95, WD 2.4 mm, water). *Z*-stacks were bidirectionally recorded across the full tissue depth of ∼2 mm with 15 µm step size.

### Image preprocessing

We corrected the *z*-axis compression resulting from the refractive index mismatch between the immersion media and the clearing media using a Fiji tool as described by Diel et al. (2020). All calculations were performed on a Dell Precision 3660 (128 GB RAM, Intel Core i9-12900, NVIDIA GeForce RTX 3070).

### TiNeQuant

The image analysis pipeline was implemented in Fiji software (NIH, Bethesda, MA, USA; Schindelin et al., 2012). The following plugins were used within the pipeline: Bio-formats importer (Linkert et al., 2010) was used for data import, LABKIT was used for image segmentation (Arzt et al., 2022), Skeletonize3D was used for 3D skeletonization. Heatmap generation was executed in Python 3.11, utilizing the data analysis library Pandas 1.5.3 (McKinney, 2010) for data preparation and GraphPad Prism 9.0.0 for visualizing the heatmap. 3D volume rendering was performed using Blender (https://www.blender.org/, accessed 12 November 2023).

### 3DVascNet

To evaluate the performance of TiNeQuant relative to existing alternative pipelines, we employed 3DVascNetV4. We installed the required cuDNN v8.1.0 and CUDA11.2 versions to utilize our NVIDIA GPU and executed the pipeline following the detailed instructions provided at https://github.com/HemaxiN/3DVascNet/wiki/Downloading-and-Running-3DVascNet. We used their pretrained classifier for segmentation and because of the high memory demand of the pipeline, exceeding our available 128 GB of RAM for complete test image stacks, we divided the image stacks for segmentation. In 3DVascNet, vessel density is defined as network area per region of interest (%), which differs from our metric, defining network density as network length per lobe volume (m/m^3^). Given that there is no possibility to extract the combined network length calculated in 3DVascNet, we calculated neuron length using our Fiji pipeline with the segmentation results acquired by 3DVascNet.

### VesselExpress

We employed VesselExpress as a second potential alternative quantification pipeline to TiNeQuant, as described in detail at https://github.com/RUB-Bioinf/VesselExpress.

### Quantification of adipocyte diameter

Transmission light microscopy was used to capture images of five randomly selected regions per sample from three iWAT samples per clearing method, following fixation and prior to the clearing procedure. Post-clearing, adipocyte autofluorescence was recorded again from five randomly selected regions within each WAT sample. We measured adipocyte diameters of 100 adipocytes per biological replicate, across the five randomly selected regions using Fiji software.

### Statistical analysis

Statistical analysis was performed in GraphPad Prism 10.4.0. To compare neuron and vascular density, 876 and 803 regions from three biological replicates per group were analyzed. ROUT outlier detection (Q=0.2%) was performed to remove statistical outliers within the dataset. For both the analysis of neuron and vascular density, as well as the adipocyte diameter measurements, a normal distribution was tested using a Shapiro–Wilk test. Subsequently, a Mann–Whitney *U*-test was performed to analyze the non-normally distributed data. We compared automated neuron length with ground-truth measurements by performing linear regression analysis and calculating the mean absolute error between the measurements.

### Large language models

ChatGPT was used to refine use of words, improve grammar and syntax of the manuscript. After using this service, the authors reviewed and edited the content as needed and take full responsibility for the content of the publication.

### Code availability

The code used in this manuscript, along with imaging data to test TiNeQuant, can be found on GitHub at https://github.com/SchweigerLab/TiNeQuant. TiNeQuant can be freely modified and distributed under the GNU-3.0 General Public License.

## Supplementary Material



10.1242/joces.263438_sup1 Supplementary information

## References

[JCS263438C1] Abadi, M., Barham, P., Chen, J., Chen, Z., Davis, A., Dean, J., Devin, M., Ghemawat, S., Irving, G., Isard, M. et al. (2016). TensorFlow: a system for large-scale machine learning. 10.48550/arXiv.1605.08695

[JCS263438C2] Alzaim, I., de Rooij, L. P. M. H., Sheikh, B. N., Börgeson, E. and Kalucka, J. (2023). The evolving functions of the vasculature in regulating adipose tissue biology in health and obesity. *Nat. Rev. Endocrinol.* 19, 691-707. 10.1038/s41574-023-00893-6 37749386

[JCS263438C3] Arzt, M., Deschamps, J., Schmied, C., Pietzsch, T., Schmidt, D., Tomancak, P., Haase, R. and Jug, F. (2022). LABKIT: Labeling and Segmentation Toolkit for Big Image Data. *Front. Comput. Sci.* 4, 777728. 10.3389/fcomp.2022.777728

[JCS263438C4] Bäckdahl, J., Franzén, L., Massier, L., Li, Q., Jalkanen, J., Gao, H., Andersson, A., Bhalla, N., Thorell, A., Rydén, M. et al. (2021). Spatial mapping reveals human adipocyte subpopulations with distinct sensitivities to insulin. *Cell Metab.* 33, 1869-1882.e6. 10.1016/j.cmet.2021.07.018 34380013

[JCS263438C5] Blaszkiewicz, M., Willows, J. W., Dubois, A. L., Waible, S., Dibello, K., Lyons, L. L., Johnson, C. P., Paradie, E., Banks, N., Motyl, K. et al. (2019). Neuropathy and neural plasticity in the subcutaneous white adipose depot. *PLoS ONE* 14, e0221766. 10.1371/journal.pone.0221766 31509546 PMC6738614

[JCS263438C6] Bonda, U., Jaeschke, A., Lighterness, A., Baldwin, J., Werner, C., De-Juan-Pardo, E. M. and Bray, L. J. (2020). 3D quantification of vascular-like structures in z stack confocal images. *STAR Protoc.* 1, 100180. 10.1016/j.xpro.2020.100180 33377074 PMC7757404

[JCS263438C7] Cao, Y., Wang, H. and Zeng, W. (2018). Whole-tissue 3D imaging reveals intra-adipose sympathetic plasticity regulated by NGF-TrkA signal in cold-induced beiging. *Protein Cell* 9, 527-539. 10.1007/s13238-018-0528-5 29589323 PMC5966360

[JCS263438C8] Chi, J., Crane, A., Wu, Z. and Cohen, P. (2018a). Adipo-clear: a tissue clearing method for three-dimensional imaging of adipose tissue. *J. Vis. Exp.* 137, 58271. 10.3791/58271 PMC6126572 30102289

[JCS263438C9] Chi, J., Wu, Z., Choi, C. H. J., Nguyen, L., Tegegne, S., Ackerman, S. E., Crane, A., Marchildon, F., Tessier-Lavigne, M. and Cohen, P. (2018b). Three-dimensional adipose tissue imaging reveals regional variation in beige fat biogenesis and PRDM16-dependent sympathetic neurite density. *Cell Metab.* 27, 226-236.e3. 10.1016/j.cmet.2017.12.011 29320703

[JCS263438C10] Corvera, S., Solivan-Rivera, J. and Yang Loureiro, Z. (2022). Angiogenesis in adipose tissue and obesity. *Angiogenesis* 25, 439-453. 10.1007/s10456-022-09848-3 35857195 PMC9519636

[JCS263438C11] Diel, E. E., Lichtman, J. W. and Richardson, D. S. (2020). Tutorial: avoiding and correcting sample-induced spherical aberration artifacts in 3D fluorescence microscopy. *Nat. Protoc.* 15, 2773-2784. 10.1038/s41596-020-0360-2 32737465

[JCS263438C12] Ertürk, A., Becker, K., Jährling, N., Mauch, C. P., Hojer, C. D., Egen, J. G., Hellal, F., Bradke, F., Sheng, M. and Dodt, H.-U. (2012). Three-dimensional imaging of solvent-cleared organs using 3DISCO. *Nat. Protoc.* 7, 1983-1995. 10.1038/nprot.2012.119 23060243

[JCS263438C13] Escobedo, N. and Oliver, G. (2017). The lymphatic vasculature: its role in adipose metabolism and obesity. *Cell Metab.* 26, 598-609. 10.1016/j.cmet.2017.07.020 28844882 PMC5629116

[JCS263438C14] Foundation, B., n.d. blender.org - Home of the Blender project - Free and Open 3D Creation Software. blender.org. URL).

[JCS263438C15] Frühbeck, G., Méndez-Giménez, L., Fernández-Formoso, J.-A., Fernández, S. and Rodríguez, A. (2014). Regulation of adipocyte lipolysis. *Nutr. Res. Rev.* 27, 63-93. 10.1017/S095442241400002X 24872083

[JCS263438C16] Haase, R., Royer, L. A., Steinbach, P., Schmidt, D., Dibrov, A., Schmidt, U., Weigert, M., Maghelli, N., Tomancak, P., Jug, F. et al. (2020). CLIJ: GPU-accelerated image processing for everyone. *Nat. Methods* 17, 5-6. 10.1038/s41592-019-0650-1 31740823

[JCS263438C17] Henriques, F., Bedard, A. H., Guilherme, A., Kelly, M., Chi, J., Zhang, P., Lifshitz, L. M., Bellvé, K., Rowland, L. A., Yenilmez, B. et al. (2020). Single-cell RNA profiling reveals adipocyte to macrophage signaling sufficient to enhance thermogenesis. *Cell Rep.* 32, 107998. 10.1016/j.celrep.2020.107998 32755590 PMC7433376

[JCS263438C18] Huesing, C., Qualls-Creekmore, E., Lee, N., François, M., Torres, H., Zhang, R., Burk, D. H., Yu, S., Morrison, C. D., Berthoud, H.-R. et al. (2021). Sympathetic innervation of inguinal white adipose tissue in the mouse. *J. Comp. Neurol.* 529, 1465-1485. 10.1002/cne.25031 32935348 PMC7960575

[JCS263438C19] Jaeschke, A., Eckert, H. and Bray, L. J. (2022). Qiber3D—an open-source software package for the quantitative analysis of networks from 3D image stacks. *GigaScience* 11, giab091. 10.1093/gigascience/giab091 35134926 PMC8848317

[JCS263438C20] Jiang, H., Ding, X., Cao, Y., Wang, H. and Zeng, W. (2017). Dense intra-adipose sympathetic arborizations are essential for cold-induced beiging of mouse white adipose tissue. *Cell Metab.* 26, 686-692.e3. 10.1016/j.cmet.2017.08.016 28918935

[JCS263438C21] Lin, Z., Chi, J. and Cohen, P. (2022). A clearing method for three-dimensional imaging of adipose tissue. *Methods Mol. Biol. Clifton NJ* 2448, 73-82. 10.1007/978-1-0716-2087-8_4 35167090

[JCS263438C22] Linkert, M., Rueden, C. T., Allan, C., Burel, J.-M., Moore, W., Patterson, A., Loranger, B., Moore, J., Neves, C., MacDonald, D. et al. (2010). Metadata matters: access to image data in the real world. *J. Cell Biol.* 189, 777-782. 10.1083/jcb.201004104 20513764 PMC2878938

[JCS263438C23] Luo, X., Jia, R., Luo, X.-Q., Wang, G., Zhang, Q., Qiao, H., Wang, N. and Yan, J. (2017). Cold exposure differentially stimulates angiogenesis in BAT and WAT of Mice: implication in adrenergic activation. *Cell. Physiol. Biochem.* 42, 974-986. 10.1159/000478680 28662501

[JCS263438C24] Martinez-Sanchez, N., Sweeney, O., Sidarta-Oliveira, D., Caron, A., Stanley, S. A. and Domingos, A. I. (2022). The sympathetic nervous system in the 21st century: neuroimmune interactions in metabolic homeostasis and obesity. *Neuron* 110, 3597-3626. 10.1016/j.neuron.2022.10.017 36327900 PMC9986959

[JCS263438C25] Massier, L., Jalkanen, J., Elmastas, M., Zhong, J., Wang, T., Nono Nankam, P. A., Frendo-Cumbo, S., Bäckdahl, J., Subramanian, N., Sekine, T. et al. (2023). An integrated single cell and spatial transcriptomic map of human white adipose tissue. *Nat. Commun.* 14, 1438. 10.1038/s41467-023-36983-2 36922516 PMC10017705

[JCS263438C26] McKinney, W. (2010). Data Structures for Statistical Computing in Python. *Proc. 9th Python Sci. Conf.*, 56-61. 10.25080/Majora-92bf1922-00a

[JCS263438C27] Narotamo, H., Silveira, M. and Franco, C. A. (2024). 3DVascNet: an automated software for segmentation and quantification of mouse vascular networks in 3D. *Arterioscler. Thromb. Vasc. Biol.* 44, 1584-1600. 10.1161/ATVBAHA.124.320672 38779855 PMC11208061

[JCS263438C28] Paszke, A., Gross, S., Massa, F., Lerer, A., Bradbury, J., Chanan, G., Killeen, T., Lin, Z., Gimelshein, N., Antiga, L. et al. (2019). PyTorch: an imperative style, high-performance deep learning library. *arXiv*, 1912.01703. 10.48550/arXiv.1912.01703

[JCS263438C29] Pirzgalska, R. M., Seixas, E., Seidman, J. S., Link, V. M., Sánchez, N. M., Mahú, I., Mendes, R., Gres, V., Kubasova, N., Morris, I. et al. (2017). Sympathetic neuron–associated macrophages contribute to obesity by importing and metabolizing norepinephrine. *Nat. Med.* 23, 1309-1318. 10.1038/nm.4422 29035364 PMC7104364

[JCS263438C30] Rauchenwald, T., Benedikt-Kühnast, P., Eder, S., Grabner, G. F., Forstreiter, S., Lang, M., Sango, R., Eisenberg, T., Rattei, T., Haschemi A. et al. (2025). Clearing the path for whole-mount labeling and quantification of neuron and vessel density in adipose tissue. *Dryad Digital Repository* 10.5061/dryad.hqbzkh1rk PMC11832183 39878039

[JCS263438C31] Renier, N., Wu, Z., Simon, D. J., Yang, J., Ariel, P. and Tessier-Lavigne, M. (2014). iDISCO: a simple, rapid method to immunolabel large tissue samples for volume imaging. *Cell* 159, 896-910. 10.1016/j.cell.2014.10.010 25417164

[JCS263438C32] Richardson, D. S., Guan, W., Matsumoto, K., Pan, C., Chung, K., Ertürk, A., Ueda, H. R. and Lichtman, J. W. (2021). Tissue clearing. *Nat. Rev. Methods Primer* 1, 84. 10.1038/s43586-021-00080-9 PMC8815095 35128463

[JCS263438C33] Robciuc, M. R., Kivelä, R., Williams, I. M., de Boer, J. F., van Dijk, T. H., Elamaa, H., Tigistu-Sahle, F., Molotkov, D., Leppänen, V.-M., Käkelä, R. et al. (2016). VEGFB/VEGFR1-induced expansion of adipose vasculature counteracts obesity and related metabolic complications. *Cell Metab.* 23, 712-724. 10.1016/j.cmet.2016.03.004 27076080 PMC5898626

[JCS263438C34] Schindelin, J., Arganda-Carreras, I., Frise, E., Kaynig, V., Longair, M., Pietzsch, T., Preibisch, S., Rueden, C., Saalfeld, S., Schmid, B. et al. (2012). Fiji: an open-source platform for biological-image analysis. *Nat. Methods* 9, 676-682. 10.1038/nmeth.2019 22743772 PMC3855844

[JCS263438C35] Schweiger, M., Schreiber, R., Haemmerle, G., Lass, A., Fledelius, C., Jacobsen, P., Tornqvist, H., Zechner, R. and Zimmermann, R. (2006). Adipose triglyceride lipase and hormone-sensitive lipase are the major enzymes in adipose tissue triacylglycerol catabolism. *J. Biol. Chem.* 281, 40236-40241. 10.1074/jbc.M608048200 17074755

[JCS263438C36] Spangenberg, P., Hagemann, N., Squire, A., Förster, N., Krauß, S. D., Qi, Y., Mohamud Yusuf, A., Wang, J., Grüneboom, A., Kowitz, L. et al. (2023). Rapid and fully automated blood vasculature analysis in 3D light-sheet image volumes of different organs. *Cell Rep. Methods* 3, 100436. 10.1016/j.crmeth.2023.100436 37056368 PMC10088239

[JCS263438C37] Vaughan, M., Berger, J. E. and Steinberg, D. (1964). Hormone-sensitive lipase and monoglyceride lipase activities in adipose tissue. *J. Biol. Chem.* 239, 401-409. 10.1016/S0021-9258(18)51692-6 14169138

[JCS263438C38] Willows, J. W., Blaszkiewicz, M., Lamore, A., Borer, S., Dubois, A. L., Garner, E., Breeding, W. P., Tilbury, K. B., Khalil, A. and Townsend, K. L. (2021). Visualization and analysis of whole depot adipose tissue neural innervation. *iScience* 24, 103127. 10.1016/j.isci.2021.103127 34622172 PMC8479257

[JCS263438C39] Willows, J. W., Blaszkiewicz, M. and Townsend, K. L. (2023). The sympathetic innervation of adipose tissues: regulation, functions, and plasticity. *Compr. Physiol.* 13, 4985-5021. 10.1002/cphy.c220030 37358505 PMC12175622

[JCS263438C40] Xie, H., Heier, C., Meng, X., Bakiri, L., Pototschnig, I., Tang, Z., Schauer, S., Baumgartner, V. J., Grabner, G. F., Schabbauer, G. et al. (2022). An immune-sympathetic neuron communication axis guides adipose tissue browning in cancer-associated cachexia. *Proc. Natl. Acad. Sci. USA* 119, e2112840119. 10.1073/pnas.2112840119 35210363 PMC8892347

[JCS263438C41] Xue, Y., Petrovic, N., Cao, R., Larsson, O., Lim, S., Chen, S., Feldmann, H. M., Liang, Z., Zhu, Z., Nedergaard, J. et al. (2009). Hypoxia-independent angiogenesis in adipose tissues during cold acclimation. *Cell Metab.* 9, 99-109. 10.1016/j.cmet.2008.11.009 19117550

[JCS263438C42] Zimmermann, R., Strauss, J. G., Haemmerle, G., Schoiswohl, G., Birner-Gruenberger, R., Riederer, M., Lass, A., Neuberger, G., Eisenhaber, F., Hermetter, A. et al. (2004). Fat mobilization in adipose tissue is promoted by adipose triglyceride lipase. *Science* 306, 1383-1386. 10.1126/science.1100747 15550674

