# Supplementary material for: Clearing the path for whole-mount labeling and quantification of neuron and vessel density in adipose tissue
Source: J Cell Sci. 2025 Feb 7;138(3):JCS263438. doi: 10.1242/jcs.263438 (PMC11832183; doi:10.1242/jcs.263438)
Supplement: Supplementary information [file joces-138-263438-s1.pdf]

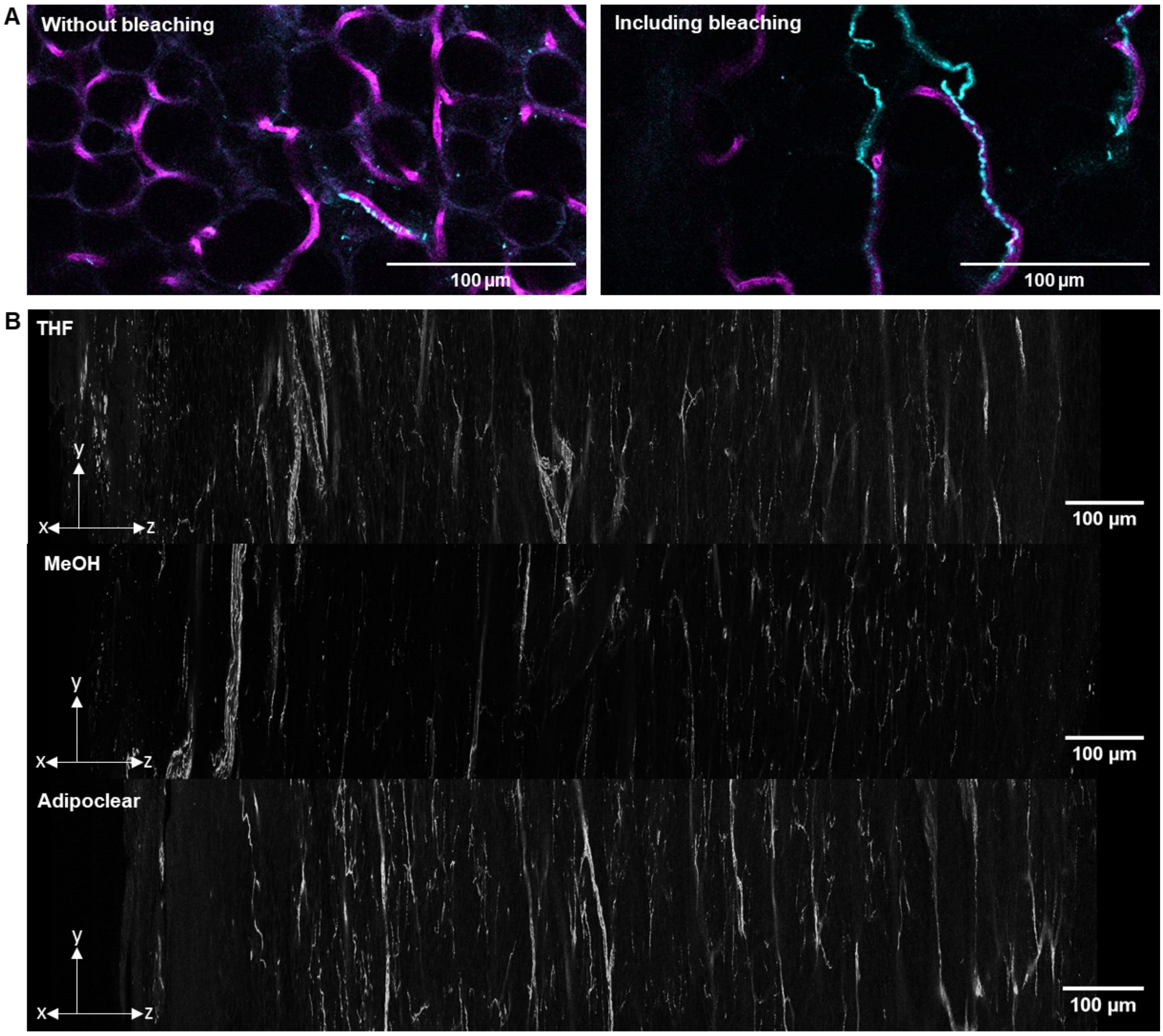

Fig. S1. Tissue autofluorescence and clarity. A Inguinal white adipose tissue (iWAT) depots were processed according to our protocol for methanol resistant antibodies (MeOH-protocol), without and including a bleaching step using hydrogen peroxide. Tissue was stained with anti-CD31 (magenta) and anti-tyrosine hydroxylase (cyan) antibodies. **B** iWAT depots were processed according to the THF-, MeOH- and Adipo-clear-protocol. 3D projections of image stacks show anti-tyrosine hydroxylase antibody signal across the z-axis, the side facing the objective is left.

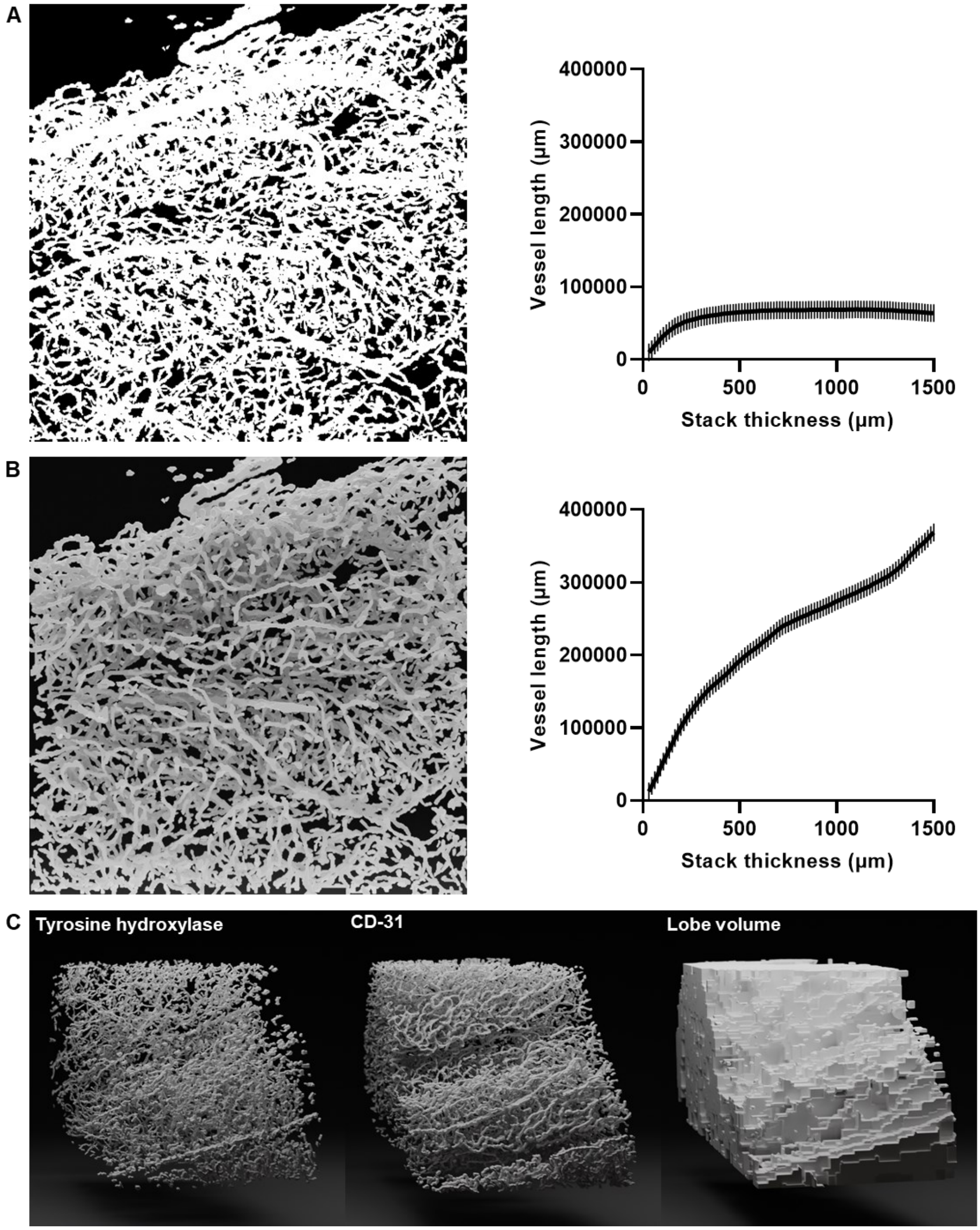

Fig. S2. The impact of increasing stack thickness on 2D and 3D network quantification and the quantification of adipose tissue lobe volume. A/B Inguinal white adipose tissue was stained with anti-CD31 antibody and vessel length quantification with increasing stack thickness in 15µm increments was performed. Each vertical line indicates one measurement. **A** 2D maximum intensity projection of the segmentation result of a 300 µm thick image stack. 2D vessel quantification in relation to increasing stack thickness. **B** 3D

volume rendering of the same image stack visualized in A. Result of 3D vessel quantification in relation to increased stack thickness. **C** The segmentation result of tyrosine hydroxylase positive sympathetic neurons and CD-31 positive vessels is combined to calculate the adipose tissue lobe volume.

Journal of Cell Science • Supplementary information

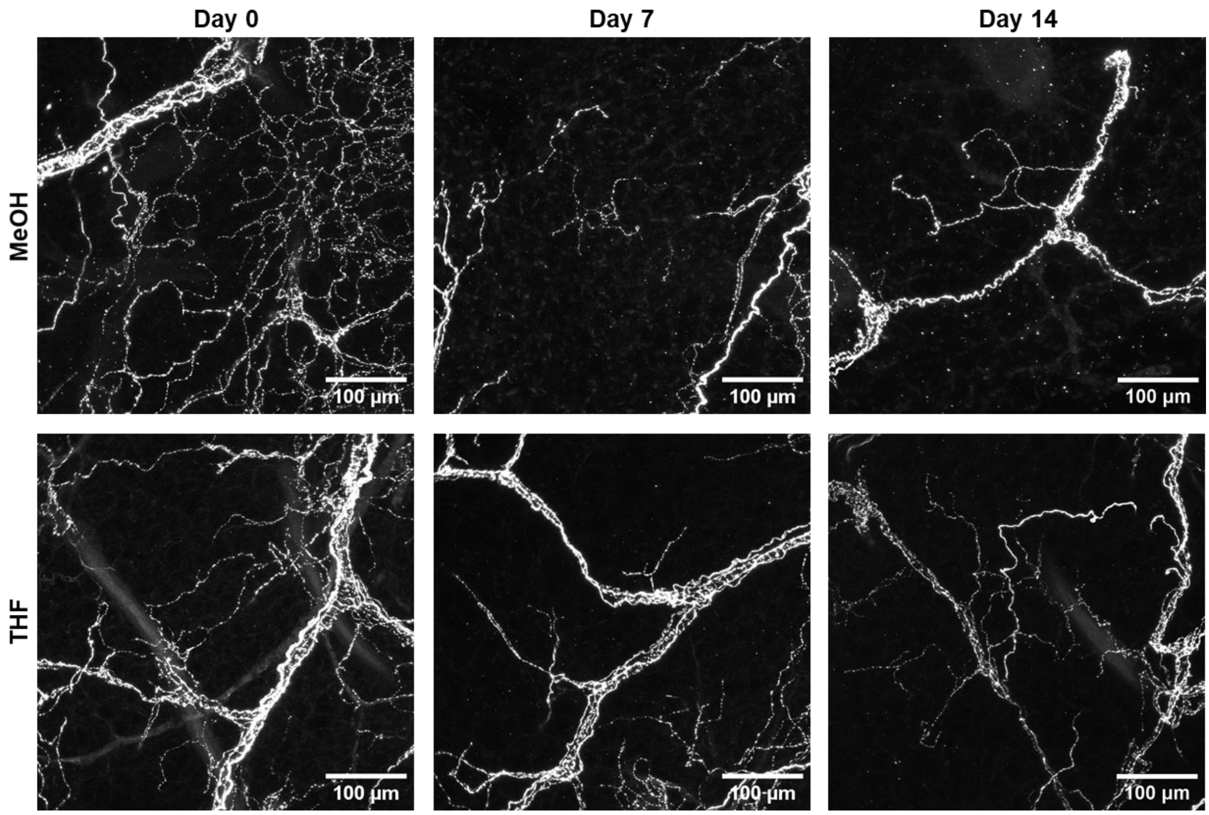

Fig. S3. Antibody labeling remains stable in both THF and MeOH clearing protocols for 14 days. Inguinal white adipose tissue samples were processed and stained with anti-tyrosine hydroxylase antibody following the MeOH- and THF-protocol. The samples were imaged directly after protocol completion (Day 0), as well as 7 days and 14 days later using identical laser and detector gain settings to those used on day 0. Images show maximum intensity projections of 150µm image stacks, all recorded at the same imaging depth.
