# Supplementary material for: Clearing the path for whole-mount labeling and quantification of neuron and vessel density in adipose tissue
Source: J Cell Sci. 2025 Feb 7;138(3):JCS263438. doi: 10.1242/jcs.263438 (PMC11832183; doi:10.1242/jcs.263438)
Supplement: Supplementary file 1 [file joces-138-263438_review_history.pdf]

Journal of Cell Science

Clearing the path for whole-mount labeling and quantification of neuron and vessel density in adipose tissue

Thomas Rauchenwald, Pia Benedikt-Kühnast, Sandra Eder, Gernot F. Grabner, Sebastian Forstreiter, Michaela Lang, Roko Sango, Tobias Eisenberg, Thomas Rattei, Arvand Haschemi, Heimo Wolinski and Martina Schweiger
DOI: 10.1242/jcs.263438

Editor: Robert Parton

Review timeline

Original submission:	16 July 2024
Editorial decision:	19 August 2024
First revision received:	6 December 2024
Accepted:	29 December 2024

Original submission

First decision letter

MS ID#: jcs.263438

MS TITLE: Clearing the path for whole-mount labeling and quantification of neuron- and vessel-density in adipose tissue

AUTHORS: Thomas Rauchenwald; Pia Benedikt-Kühnast; Sandra Eder; Roko Sango; Thomas Rattei; Arvand Haschemi; Heimo Wolinski; Martina Schweiger

ARTICLE TYPE: Tools and Resources

Dear Dr Schweiger,

We have now reached a decision on the above manuscript.

To see the reviewers' reports and a copy of this decision letter, please go to:

The reviewers raise a number of substantial criticisms that prevent me from accepting the paper. As you will see, one of the reviewers felt that there were limited technical advances and that to make the manuscript suitable for publication in the Journal of Cell Science one option would be to address the biological questions in more detail. The second reviewer also raised a number of significant technical concerns, although they did suggest that a revised manuscript might be able to address their concerns.

After some deliberation, I have decided that the fairest course of action is to give you the chance to address these concerns in a revised manuscript. However, I realise that substantial revisions are required to answer the concerns of both reviewers and that you might want to consider other publication options (including transfer to biology Open). If you do choose to resubmit a revised manuscript please ensure you address all the comments as I will need to return it to the reviewers.

Comments from the Reviewers:

© 2025. Published by The Company of Biologists under the terms of the Creative Commons Attribution License (https://creativecommons.org/licenses/by/4.0/).

Reviewer 1: In their manuscript "Clearing the path for whole-mount labeling and quantification of neuron- and vessel-density in adipose tissue" Rauchenwald et al., outline a method for clearing adipose tissue and quantifying neuron and blood vessel density within the tissue. The paper is submitted as a "Tools and Resources" article suggesting its focus is method development; however, the authors also attempt to answer a biological question of whether the nervous and/or circulatory systems in adipose tissue are remodeled in response to heat or cold. Currently, I find the study to be too preliminary to be ready for publication. My justification for this conclusion is:
The authors do not directly compare their methods to current gold standards. This is an essential control experiment for any new methods paper. On the clearing side, the gold standard would be the AdipoClear method. The authors should show and quantify a) the overall clarity of the tissue and b) the quality of labeling. For the quantification of neuron and blood vessel density, there are many options. Here are a few of the most recent that could be used for comparison: 3DVascNet (3DVascNet: An Automated Software for Segmentation and Quantification of Mouse Vascular Networks in 3D, https://doi.org/10.1161/ATVBAHA.124.320672); Vessel Express (Rapid and fully automated blood vasculature analysis in 3D light-sheet image volumes of different organs, https://doi.org/10.1016/j.crmeth.2023.100436); MeasureNeurons module in Cell Profiler, neuTube (neuTube 1.0: A New Design for Efficient Neuron Reconstruction Software Based on the SWC Format, https://doi.org/10.1523/ENEURO.0049-14.2014); or the Consensus algorithm of the BigNeuron project (BigNeuron: a resource to benchmark and predict performance of algorithms for automated tracing of neurons in light microscopy datasets, https://doi.org/10.1038/s41592-024-02395-3). Here, the authors should not only quantify accuracy to ground truth, but also speed and maximum data set size.

As the methods (both clearing and image quantitation) only show minor changes to currently accepted practices, unless the direct comparisons suggested above show vast improvements, I would recommend the authors expand on the biology side of the story - which seems promising, interesting, and able to answer a debate in the field - and consider a standard (not Tools and Resources) submission.

Other minor comments:
1) The THF gradient is quite harsh (50%, 75%, 100%) the authors may want to consider, or comment, that a gentler gradient will better preserve the morphology of the tissue.
2) The authors use a water immersion objective calibrated to RI = 1.33 to image tissue in DBE (RI = 1.56). This will introduce extensive spherical aberration. Was this corrected for while imaging? (see: Tutorial: avoiding and correcting sample-induced spherical aberration artifacts in 3D fluorescence microscopy https://doi.org/10.1038/s41596-020-00459-0)
3) Figure 4 needs to show a representative image of neuron and vascular staining for mice treated at 4 and 30 C. Currently, they only show neuronal staining and do not indicate which temperature treatment was performed in the figure legend.
4) In Figure 5b - does the heat map of "neuron length per regional volume" give any more information than a quantitation of # of pixels over a certain intensity threshold in each regional volume? Would this be less computationally intensive?

Reviewer 2: SUMMARY OF THE ADVANCE MADE IN THIS PAPER AND ITS POTENTIAL SIGNIFICANCE TO THE FIELD

This is an important new contribution to the body of literature and methods that assess nerve content in adipose tissues. As more and more findings clearly indicate the relevance and importance of adipose innervation, being able to appropriately and accurately quantify tissue axon density and distribution will be even more important. Approachable, open-access methodologies able to be applied across numerous lab settings will be important. This group has added an additional clearing method that can expand antibody options, and provided a Fiji-based quantification approach. However, numerous concerns exist with the presentation of this new approach, as outlined below, and should be addressed before publication.

SUGGESTIONS TO AUTHORS
1) A major concern in this novel method is that the authors are not seeing small fiber axons, which are most likely to change with neuropathy or cold-induced neuroplasticity...perhaps the approach or Ab are not ideal to see small fibers. Also adding a TN group in addition to RT and cold is

© 2025. Published by The Company of Biologists under the terms of the Creative Commons Attribution License (https://creativecommons.org/licenses/by/4.0/).

important for assessing nerve remodeling. However, the vascular imaging is gorgeous and should be lauded.
2) An unfortunate caveat of their approach is the limited stability of the samples for imaging-(lines 152-153, and 159);
"The samples can be stored at 4°C and remain stable for imaging for at least 5 days." Whole depot imaging of a single animal can take overnight, so this is challenging for cohort studies
3) Claims at lines 80-82 in the Intro and lines 373-7 of Discussion - Important distinctions among these papers: some use TH (mainly sympathetic, but in adipose and numerous other tissues also marks a large proportion of sensory), some use pan-neuronal markers. In addition, whole-depot/tissue quantification varies between animals, and across the tissue there is high regional variation - the beige islets/lymph node regions have much more branching/outgrowth with cold, especially if using TH as a marker - this seems to be consistent across studies. As written, the authors have misinterpreted the existing data and the nuance needs to be clarified.
In addition, the claims in Discussion (338-40 lines) appear to be inaccurate - it looks like this paper showed images not reconstructed in 3D, but a series of 2D images in tiled z-stacks quantified across the entire 3-dimensional tissue. Whole tissue max projections were shown as representative but do not appear to have been used for quantification.
4) the sole use of TH in this study is additionally confounded, because not only does it mark axons, but TH levels increase when nerves are activated (such as with cold).
5) in Lines 208-9 the authors claim adipose morphology is preserved with this approach - this is not demonstrated and is tough to believe. Clearing methods are well known to affect tissue morphology (swelling, distortion) and this is not adequately addressed; even if it is a caveat it would not discount the method and should be honestly discussed. For example, Disco-approaches are known to shrink and harden tissues.

First revision

Author response to reviewers' comments

Dear Reviewers,
We are thankful for the opportunity and pleased to re-submit our revised manuscript entitled “Clearing the path for whole-mount labelling and quantification of neuron- and vessel-density in adipose tissue” by Thomas Rauchenwald et al. for consideration for publication in the Journal of Cell Science.
We appreciate the time and consideration you have been given to our work. Incorporating the revisions significantly enhanced the quality of the manuscript. We addressed all the comments raised in the point-to-point response below.

Point to point response to the reviewers:

Reviewer 1:
In their manuscript "Clearing the path for whole-mount labeling and quantification of neuron- and vessel-density in adipose tissue" Rauchenwald et al., outline a method for clearing adipose tissue and quantifying neuron and blood vessel density within the tissue. The paper is submitted as a "Tools and Resources" article suggesting its focus is method development; however, the authors also attempt to answer a biological question of whether the nervous and/or circulatory systems in adipose tissue are remodeled in response to heat or cold. Currently, I find the study to be too preliminary to be ready for publication. My justification for this conclusion is: The authors do not directly compare their methods to current gold standards. This is an essential control experiment for any new methods paper. On the clearing side, the gold standard would be the AdipoClear method. The authors should show and quantify a) the overall clarity of the tissue and b) the quality of labeling.

We thank reviewer 1 for the comprehensive review and constructive comments which have been instrumental in improving our manuscript. Our goal is to provide the scientific community an easy-to-use method for quantifying network structures in whole mount image-stacks. We have revised the manuscript to emphasize the methodological aspect more

© 2025. Published by The Company of Biologists under the terms of the Creative Commons Attribution License (https://creativecommons.org/licenses/by/4.0/).

prominently, presenting the biological aspects as proof of principle, as required by journal guidelines. To demonstrate the possibilities our improved method for network quantification offers to answer biological questions we show the density of the vasculature and nerves of adipose tissue under thermoneutral and cold conditions. We have now directly compared our tissue-clearing approach with the current gold standard, Adipo-clear. In this comparison between Adipo-clear and both our methanol-containing and methanol-free tissue-clearing methods, we observed no discernible differences in labeling quality (**Attached Figure 1 A**) or tissue clarity (**Attached Figure 1 B**). However, we found that our adipose tissue clearing approach minimizes adipocyte shrinkage. While we did not find significant changes in adipocyte size using our methanol free protocol (THF), we observed significant adipocyte shrinkage using the methanol containing protocol (-2.8%) and Adipo-clear (-15.0%) (**Attached Figure 1 C**). We also found that certain antibodies or the epitopes thereof, are methanol-sensitive. While methanol-based protocols, including Adipo-clear proved ineffective with methanol-sensitive antibodies our THF protocol proved to be successful (**Attached Figure 1 D**). We show these new results in **Figure 1** and **Supplementary Figure 1 C** of the revised manuscript.

© 2025. Published by The Company of Biologists under the terms of the Creative Commons Attribution License (https://creativecommons.org/licenses/by/4.0/).

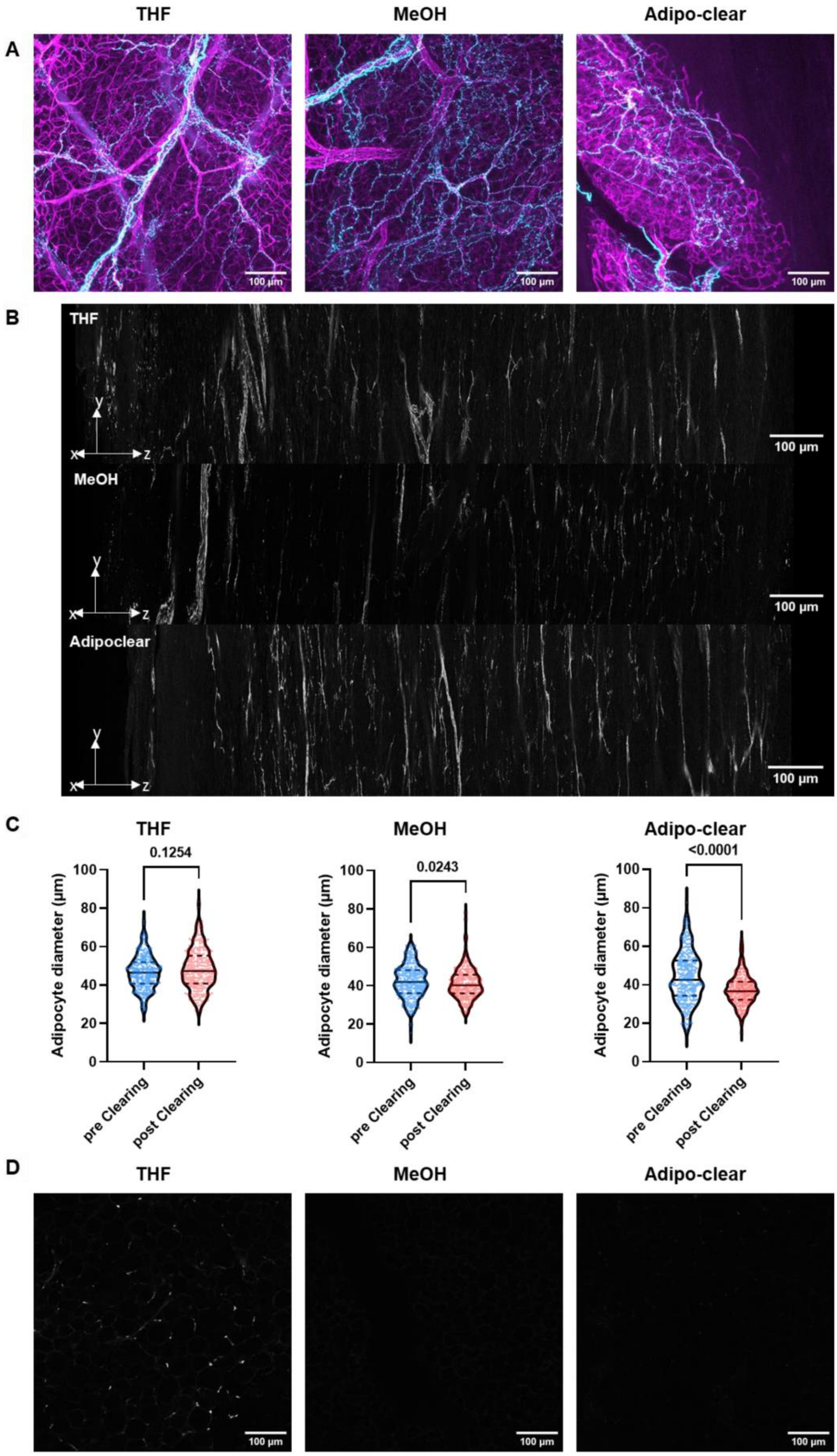

Attached Figure 1: A Inguinal white adipose tissue (iWAT) depots stained with anti-tyrosine hydroxylase (cyan) and anti-CD31 (magenta) antibodies, processed with the THF-protocol, the

© 2025. Published by The Company of Biologists under the terms of the Creative Commons Attribution License (https://creativecommons.org/licenses/by/4.0/).

MeOH-protocol, or Adipo-clear. **B** 3D projections of image stacks show anti-tyrosine hydroxylase antibody signal in iWAT depots across the z-axis, the side facing the objective is left. **C** Violin plots with median (solid line) and quartiles (dashed lines) showing adipocyte diameter before and after iWAT clearing with the THF-protocol, the MeOH-protocol or Adipo-clear. 300 adipocytes across five randomly selected regions in three biological replicates per group were measured. A Shapiro-Wilk test was performed to test for normal distribution, then a Mann-Whitney U test was performed to analyze the non-normally distributed data. **D** iWAT depots stained with methanol sensitive anti-CD- 68 antibody processed with the THF-protocol for methanol sensitive antibodies, the MeOH-protocol for methanol resistant antibodies or Adipo-clear, images were recorded using identical settings and imaging depth.

For the quantification of neuron and blood vessel density, there are many options. Here are a few of the most recent that could be used for comparison: 3DVascNet (3DVascNet: An Automated Software for Segmentation and Quantification of Mouse Vascular Networks in 3D, https://doi.org/10.1161/ATVBAHA.124.320672); Vessel Express (Rapid and fully automated blood vasculature analysis in 3D light-sheet image volumes of different organs, https://doi.org/10.1016/j.crmeth.2023.100436); MeasureNeurons module in Cell Profiler, neuTube (neuTube 1.0: A New Design for Efficient Neuron Reconstruction Software Based on the SWC Format, https://doi.org/10.1523/ENEURO.0049-14.2014); or the Consensus algorithm of the BigNeuron project (BigNeuron: a resource to benchmark and predict performance of algorithms for automated tracing of neurons in light microscopy datasets, https://doi.org/10.1038/s41592-024-02395-3). Here, the authors should not only quantify accuracy to ground truth, but also speed and maximum data set size.

We thank the reviewer for this advice and for the provided examples of network quantification software for the comparison with TiNeQuant. We compared our TiNeQuant pipeline to 3DVascNet and VesselExpress since these seemed to be the most applicable pipelines for whole mount network quantification to us. We performed linear regression analysis on the outcome by plotting ground truth neuron lengths against the automatically calculated neuron lengths for each of our test stacks. The level of accuracy was much improved using TiNeQuant (Mean absolute error=33.2mm) (**Attached Figure 2 A**) in comparison with VesselExpress (Mean absolute error=60.2mm) (Attached Figure 2 B) and 3DVascNet (Mean absolute error=84.3) (**Attached Figure 2 C**). The results of the manual quantification of 6 independent annotators were similar to TiNeQuant (absolute error to mean =2.1mm), while in comparison VesselExpress (absolute error to mean =11.0mm) and 3DVascNet (absolute error to mean=12.6mm) demonstrated decreased accuracy (**Attached Figure 2 D**). We also measured the computation time using the test dataset used in Attached Figure A-C and observed that, while VesselExpress was 29% slower than TiNeQuant, 3DVascNet required 654 times longer to process this dataset (**Attached Figure 2 E**). This means that the estimated calculation time for processing an average whole mount scanned adipose tissue depot using 3DVascNet is 1740 h 6 min, while VesselExpress takes 3 h 26 min, TiNeQuant completes the same operation in 2 h 40 min. We show these new results in **Figure 4** of the revised manuscript. We also measured maximum processable dataset size, which is most likely limited by RAM. We used the same test stack to measure peak RAM usage, which peaked at 3 GB for TiNeQuant, 5 GB for VesselExpress and 95 GB for 3DVascNet. Clearly the quantification of large 3D networks surpasses the intended use of 3DVascNet for retinal vascular networks at the current stage. This information is provided in the discussion section of the revised manuscript: *"One further consideration is the maximum processable dataset size, which is most limited by RAM usage. For a test stack of 37 images, measuring 512 x 512 pixels, the peak RAM usage was 3 GB using TiNeQuant, 5 GB for VesselExpress and 95 GB for 3DVascNet."*

© 2025. Published by The Company of Biologists under the terms of the Creative Commons Attribution License (https://creativecommons.org/licenses/by/4.0/).

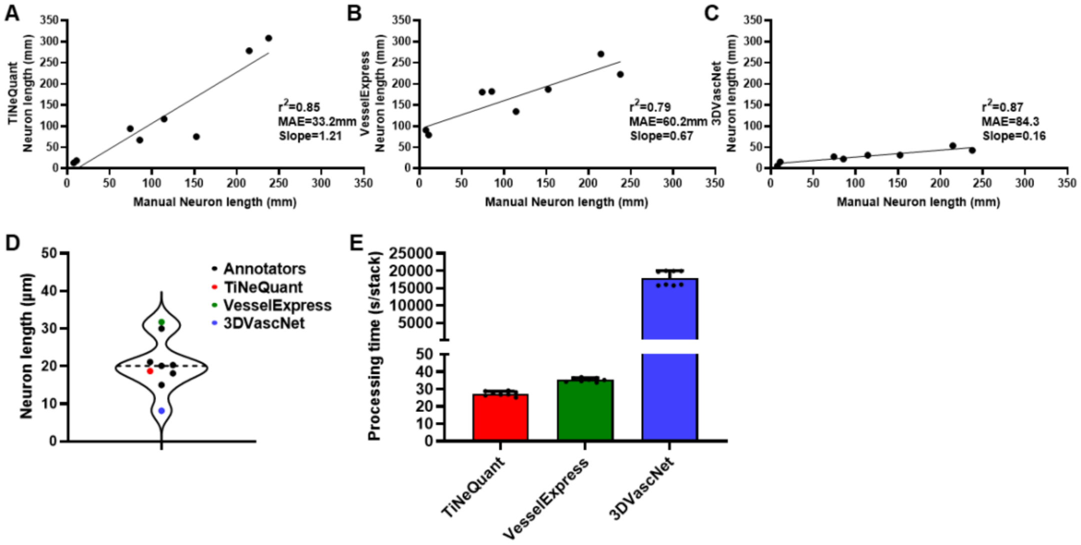

Attached Figure 2: Inguinal white adipose tissue (iWAT) depots excised from mice housed at 30°C or 4°C for seven days were processed with our methanol containing (MeOH) clearing protocol. Image stacks of anti-tyrosine hydroxylase antibody labeled neurons were selected evenly between the two housing conditions and randomly selected across the whole depot of three biological replicates. **A/B/C** Linear regression analysis of manual neuron length quantification plotted against automated neuron length quantification by TiNeQuant (A), Vessel Express (B) and 3DVascNet (C). **D** Accuracy was tested by comparing the results obtained from 6 independent manual annotators with the results of the automated quantification by our pipeline. **E** Processing time per imagestack of TiNeQuant, VesselExpress and 3D VascNet (test dataset contained 8 (n=8) image stacks with a total of 1124 512 x 512 pixels slices). Graph shows mean+s.d.

As the methods (both clearing and image quantitation) only show minor changes to currently accepted practices, unless the direct comparisons suggested above show vast improvements, I would recommend the authors expand on the biology side of the story - which seems promising, interesting, and able to answer a debate in the field - and consider a standard (not Tools and Resources) submission.

We believe that due to the significant improvements regarding antibody usability, accessibility by excluding transcardiac perfusion and improved preservation of tissue morphology our protocols provide a meaningful addition to the current adipose tissue clearing toolbox. Atop TiNeQuant is easy to use via its user interface and accurate. Classifier training can easily be done within minutes on CPUs of consumer grade computers providing tailor made segmentation capabilities. This democratizes robust 3D Network quantification and is a meaningful contribution to future efforts studying tissue network density. We agree that the results on the differences in vasculature between thermoneutral and cold-housed animals are very interesting and definitively deserve further investigations. In this manuscript however, we aim to equip the scientific community with improved resources to study innervation and vasculature under different conditions.

Other minor comments:

1) The THF gradient is quite harsh (50%, 75%, 100%) the authors may want to consider, or comment, that a gentler gradient will better preserve the morphology of the tissue.

We agree with the reviewer and included following statement in the methods section of the revised manuscript:

"A gentler gradient with shorter incubation times may preserve the tissue morphology even better."

2) The authors use a water immersion objective calibrated to RI = 1.33 to image tissue in DBE (RI = 1.56). This will introduce extensive spherical aberration. Was this corrected for while imaging? (see: Tutorial: avoiding and correcting sample-induced spherical aberration artifacts in 3D fluorescence microscopy https://doi.org/10.1038/s41596-020-00459-0)

© 2025. Published by The Company of Biologists under the terms of the Creative Commons Attribution License (https://creativecommons.org/licenses/by/4.0/).

We are thankful for this thoughtful suggestion, as it indeed improved the accuracy of the absolute measured network lengths. Using the recommended macro from the suggested publication we found that the z-axis compression caused by this refractive index mismatch induced an absolute reduction of network length of 15- 25%. We redid all the quantifications with the corrected data and found that while the absolute network length increases after the correction, of course so did the calculated lobe volume. While improving the absolute correctness of calculated network length we only saw minor changes in the measured network density between the quantification of network length without RI mismatch correction (**Attached Figure 3 A**) and including the RI mismatch correction as seen in the revised manuscript (**Attached Figure 3 B and Figure 5C** of the revised manuscript).

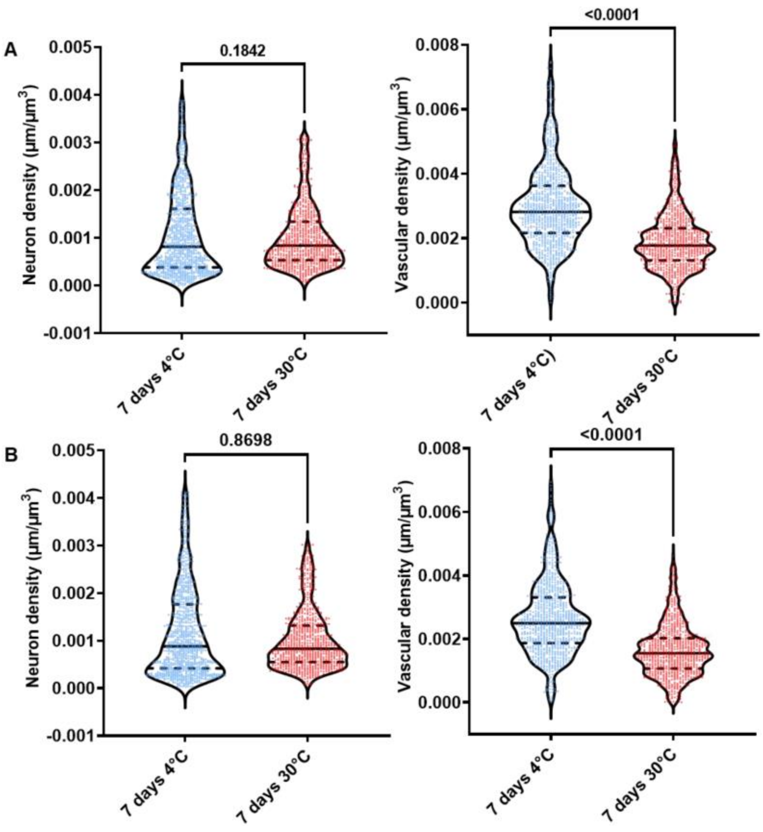

Attached Figure 3: C57BL/6J mice were either single-housed at 30°C or at 4°C for 7 days. Mice were sacrificed, inguinal white adipose tissue excised, treated according to the protocol for methanol-insensitive antibodies and immunolabeled using anti- tyrosine hydroxylase and anti-CD31 antibody. iWAT neuron and vascular density was measured across approximately 850 regions and three biological replicates per group. **A** Quantification without z-axis compression compensation. **B** Quantification including z-axis compression compensation. A Shapiro-Wilk test was performed to test for normal distribution, then a Mann-Whitney U test was performed to analyze the non-normally distributed data. Violin plots display the median (solid line) and quartiles (dashed lines).

3) Figure 4 needs to show a representative image of neuron and vascular staining for mice treated at 4 and 30 C. Currently, they only show neuronal staining and do not indicate which temperature treatment was performed in the figure legend.

Thank you for this comment, we absolutely agree that we need to show representative images of the two conditions. Since figure 4 does not directly compare cold-exposed and thermoneutral kept mice but only shows the evaluation of our quantification pipeline, we added the representative images of neuron and vascular stainings for mice housed at 4°C and 30°C to Figure 5 (**Attached Figure 4 and Figure 5D** in the revised manuscript), we believe that this way the storyline is easier to understand. We matched the chosen examples as

© 2025. Published by The Company of Biologists under the terms of the Creative Commons Attribution License (https://creativecommons.org/licenses/by/4.0/).

closely as possible to the mean neuron, and vascular density (Neuron density 7 days 4°C mean: 0.00117 m/m3 example:
0.00116 m/m3, 7 days 30°C mean: 0.00102 m/m3 example: 0.00102 m/m3, Vascular density 7 days 4°C mean: 0.00265 m/m3 example: 0.00268 m/m3, 7 days 30°C mean: 0.00164 m/m3 example: 0.00165 m/m3).

We are sorry for having missed to included the information about the temperature treatment and added the missing sample information to the figure legends of **Figure 4 and 5.***"**Figure 4: Evaluation of automated neuron length quantification**. Inguinal white adipose tissue (iWAT) depots excised from mice housed at 30°C or 4°C for seven days were processed with our methanol containing (MeOH) clearing protocol. Image stacks of anti-tyrosine hydroxylase antibody labeled neurons were selected evenly between the two housing conditions and randomly selected across the whole depot of three biological replicates."*"***Figure 5: Analyzing sympathetic innervation and vascular density in iWAT of mice housed at different ambient temperatures**. C57BL/6J mice were either single- housed at 30°C or at 4°C for seven days. Mice were sacrificed, inguinal white adipose tissue (iWAT) excised, treated according to the protocol for methanol-insensitive antibodies and immunolabeled using anti-tyrosine hydroxylase and anti-CD31 antibody. **A** Exemplary maximum intensity projection of an anti-tyrosine hydroxylase immunolabeled and whole-mount cleared iWAT sample of a mouse housed at 4°C. **B** Heatmap showing measured neuron density (neuron length per regional lobe volume) of the same sample."*

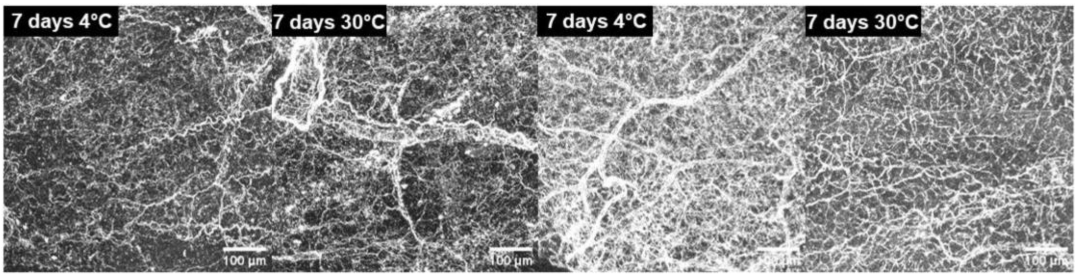

Attached Figure 4: C57BL/6J mice were either single-housed at 30°C or at 4°C for seven days. Mice were sacrificed, inguinal white adipose tissue (iWAT) excised, treated according to the protocol for methanol-insensitive antibodies and immunolabeled using anti-tyrosine hydroxylase and anti-CD31 antibody. Representative maximum intensity projections of single tiles that were used for quantification. The chosen examples match the mean neuron, and vascular density (Left: neuron density 7 days 4°C mean: 0.00117 m/m3 example: 0.00116 m/m3, 7 days 30°C mean: 0.00102 m/m3 example: 0.00102 m/m3, right: vascular density 7 days 4°C mean: 0.00265 m/m3 example: 0.00268 m/m3, 7 days 30°C mean: 0.00164 m/m3 example: 0.00165 m/m3).

4) In Figure 5b - does the heat map of "neuron length per regional volume" give any more information than a quantitation of # of pixels over a certain intensity threshold in each regional volume? Would this be less computationally intensive?

We thank the reviewer for the constructive input. We did a comparison and tested the correlation between ground truth neuron length and the area of pixels across the image stack above a fixed threshold that fitted the neuron signal the best (8-bit gray value>170) (**Attached Figure 5 and Figure 4D** in the revised manuscript). While being computationally inexpensive, the so calculated thresholded area showed to be a poor predictor of neuron length (r2=0.28). This is probably since very large neurons, while showing a great area above the threshold, when skeletonized they don´t add more to the neuron density than a thin neuron sprout. We believe that for the application of measuring network sprouting, neuron density ($\mu m/\mu m^3$) is the most expressive measurement.

© 2025. Published by The Company of Biologists under the terms of the Creative Commons Attribution License (https://creativecommons.org/licenses/by/4.0/).

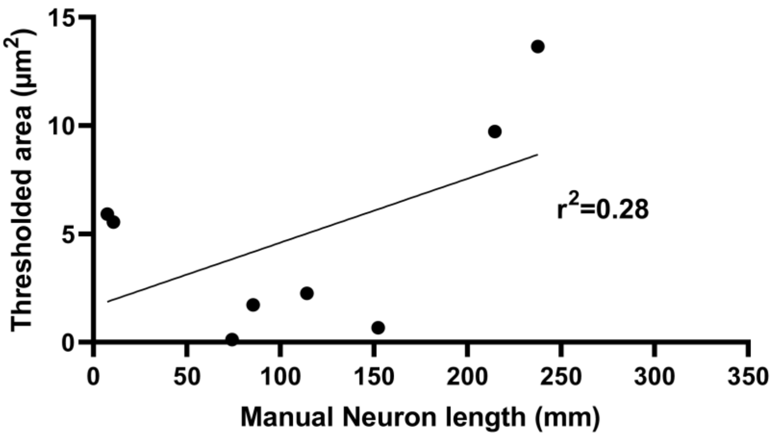

Attached Figure 5: Inguinal white adipose tissue (iWAT) depots excised from mice housed at 30°C or 4°C for seven days were processed with our methanol containing (MeOH) clearing protocol. Image stacks were selected evenly between the two housing conditions and randomly selected across the whole depot of three biological replicates. Linear regression analysis of manual neuron length quantification plotted against the thresholded pixel area (8-bit gray value>170).

Reviewer 2: SUMMARY OF THE ADVANCE MADE IN THIS PAPER AND ITS POTENTIAL SIGNIFICANCE TO THE FIELD

This is an important new contribution to the body of literature and methods that assess nerve content in adipose tissues. As more and more findings clearly indicate the relevance and importance of adipose innervation, being able to appropriately and accurately quantify tissue axon density and distribution will be even more important. Approachable, open-access methodologies able to be applied across numerous lab settings will be important. This group has added an additional clearing method that can expand antibody options, and provided a Fiji-based quantification approach. However, numerous concerns exist with the presentation of this new approach, as outlined below, and should be addressed before publication.

SUGGESTIONS TO AUTHORS

1) A major concern in this novel method is that the authors are not seeing small fiber axons, which are most likely to change with neuropathy or cold-induced neuroplasticity...perhaps the approach or Ab are not ideal to see small fibers.

We thank Reviewer 2 for the time invested in the careful review and in improving our work. We agree that it is still possible that we miss sensitivity to resolve even smallest axons. However, with all the testing regarding antibodies against pan neuronal, sympathetic neuronal, or neuron growth associated epitopes anti-tyrosine hydroxylase in our hands turned out to be by far the most sensitive option. Since tyrosine hydroxylase protein levels increase during cold exposure it is critical to have highest possible sensitivity to avoid the confounding effect of higher neuron detectability due to increased protein expression. The fact that we did not detect an increase in neuron density in cold-exposed mice indicates that we achieved a sensitivity level sufficient to already detect very small axons. Yet the better is the enemy of the good and it is thinkable that future, even more sensitive approaches may challenge our current views. We made this controversy clear in the discussion: *"In our hands, of all the antibodies tested, TH was the most sensitive option for staining sympathetic neurons. This is crucial, as axonal outgrowth predominantly affects only the smallest neurons. One major caveat is that TH levels rise during cold exposure, so highly sensitive imaging is essential to avoid confusion from increased neuron detectability caused by higher protein expression. The fact that we did not detect increased neuron density in cold-exposed mice indicates that we achieved a sensitivity level sufficient to detect the smallest axons which are most likely to change during metabolic adaptation. In comparison to light sheet microscopy (Cao et al., 2018; Xie et al., 2022), the use of confocal laser scanning microscopy, as employed in our study and by others (Blaszkiewicz et al., 2019; Willows et al., 2021), allows higher*

© 2025. Published by The Company of Biologists under the terms of the Creative Commons Attribution License (https://creativecommons.org/licenses/by/4.0/).

sensitivity and resolution and leads to the capture of finer structures. However, it is thinkable that future, even more sensitive approaches may challenge our current views."

Also adding a TN group in addition to RT and cold is important for assessing nerve remodeling. However, the vascular imaging is gorgeous and should be lauded.

We are sorry for not pointing out clear enough that our study indeed compares cold exposed mice (7 days at 4°C) with mice kept at thermoneutral conditions (7 days at 30°C) in order to observe a maximum effect on remodeling. We now put more emphasis on the temperature differences in the revised manuscript. We agree that the results on the differences in vasculature between thermoneutral and cold-housed animals are very interesting and definitively deserve further investigations. This is however not our expertise and beyond the scope of this manuscript. Here, with TiNeQuant, we aim to equip the scientific community with a feasible tool to study innervation and vasculature under different conditions.

2) An unfortunate caveat of their approach is the limited stability of the samples for imaging-(lines 152-153, and 159);
"The samples can be stored at 4°C and remain stable for imaging for at least 5 days." Whole depot imaging of a single animal can take overnight, so this is challenging for cohort studies

We thank the reviewer for this comment. We have taken the feedback to heart and tested the stability of our samples. Upon monitoring signal degradation over a time span of 14 days we did not observe any signal decay or morphologic changes (**Attached Figure 6**). Even samples that were stored for several months (not shown) didn't show any obvious signs of signal degradation, so presumably the samples can easily be imaged at even later timepoints post clearing. We added the following statement to the revised manuscript: "The samples can be stored at 4°C and remain stable for imaging for at least 14 days without signal deterioration (Fig. S3)." and added the images as **Supplemental Figure 3 (Attached Figure 6).**

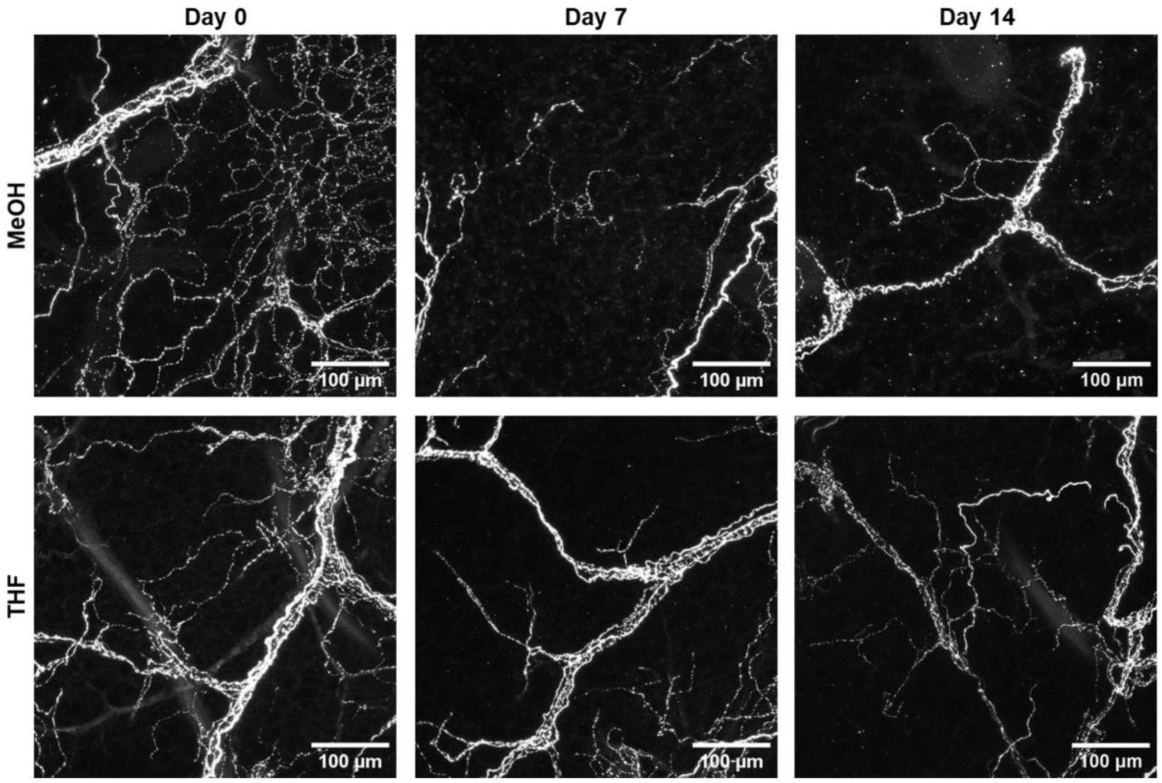

Attached Figure 6: Inguinal white adipose tissue samples were processed and stained with anti-tyrosine hydroxylase antibody following the MeOH- and THF-protocol. The samples were imaged directly after protocol completion (Day 0), as well as 7 days and 14 days later using

© 2025. Published by The Company of Biologists under the terms of the Creative Commons Attribution License (https://creativecommons.org/licenses/by/4.0/).

identical laser and detector gain settings to those used on day 0. Images show maximum intensity projections of 150µm image stacks, all recorded at the same imaging depth.

3) Claims at lines 80-82 in the Intro and lines 373-7 of Discussion - Important distinctions among these papers: some use TH (mainly sympathetic, but in adipose and numerous other tissues also marks a large proportion of sensory), some use pan-neuronal markers. In addition, whole-depot/tissue quantification varies between animals, and across the tissue there is high regional variation - the beige islets/lymph node regions have much more branching/outgrowth with cold, especially if using TH as a marker - this seems to be consistent across studies. As written, the authors have misinterpreted the existing data and the nuance needs to be clarified.

We thank the reviewer for pointing out these aspects and discrepancies between different studies. Addressing them improved the revised manuscript. We now address the complexity of this matter in more detail. We absolutely agree that antibody selection is crucial and that capturing the regional variation is essential. Hence, we propagate whole mount imaging and unbiased global density quantification. As already discussed above, in our hands, of all the antibodies tested, tyrosine hydroxylase was the most sensitive option but we agree that only using this neuron specific antibody does not resolve the whole debate. In our view this manuscript adds a small piece to this exciting puzzle and we hope that our tissue clearing and quantification approach helps the scientific community to solve the puzzle.

We extended the Introduction and Discussion section and now write:

Introduction: *"While some studies argue for a substantial adaptability in sympathetic neuron density, thereby regulating the sympathetic stimulus during cold adaptation in WAT (Cao et al., 2018), these findings could not be affirmed by other researchers (Blaszkiewicz et al., 2019; Willows et al., 2021). Notably differences in outcome can be based on antibody sensitivity and specificity, imaging resolution and regional variability of sympathetic innervation across the tissue."*

Discussion: *"Previous studies suggested that enhanced intraadipose sympathetic activity is due to axon outgrowth in cold-exposed and cachectic animals (Cao et al., 2018; Xie et al., 2022). In these approaches, random WAT regions were selected for quantification. Due to the heterogeneous distribution of sympathetic neurons in adipose tissue, sole quantification of a randomly selected subfraction of the tissue may not be sufficient for reliable comparative analyses. In contrast, our approach setting neuron length in relation to local lobe volume provides information about spatial heterogeneity and allows robust global density quantification in the whole tissue sample in an automated and unbiased manner. Performing whole mount analysis, we did not observe a global increase in sympathetic neuron density in WAT upon cold exposure, which is consistent with recent findings (Blaszkiewicz et al., 2019; Willows et al., 2021). It must be noted that Blaszkiewicz et al. and Willows et al. used the pan-neuronal markers PGP9.5 and beta-3 tubulin, while Cao et al. and Xie et al. used pan-neuronal (Synaptophysin), mostly sympathetic neuron-specific (TH) markers as well as Stathmin-2 and Growth-associated protein-43 as markers for axon outgrowth."*

In addition, the claims in Discussion (338-40 lines) appear to be inaccurate - it looks like this paper showed images not reconstructed in 3D, but a series of 2D images in tiled z-stacks quantified across the entire 3-dimensional tissue. Whole tissue max projections were shown as representative but do not appear to have been used for quantification.

The reviewer addresses an important point, however, we have again taken a close look at the methodology section of the paper referred to (Willows et al. 2021) and have extracted the following sections: *"These tiles were then merged and processed into a 2D maximum intensity projection either in LASX (Leica imaging software) or in Fiji (Schindelin et al., 2012) if being used for quantification"*; *"Maximum intensity projections through the Z- dimension (Z-max projection) were generated for each tile individually using Fiji (Schindelin et al., 2012)."* and *"Except for an experimental analysis of 2D Z-max projections vs. the individual 2D slices making up the 3D confocal data (Figure S5 in Willows et al., 2021), total nerve*

© 2025. Published by The Company of Biologists under the terms of the Creative Commons Attribution License (https://creativecommons.org/licenses/by/4.0/).

length was calculated using values measured for each 2D Z- max projection." This information points towards 2D-image quantification of nerve length.

4) the sole use of TH in this study is additionally confounded, because not only does it mark axons, but TH levels increase when nerves are activated (such as with cold).

We thank the reviewer for highlighting the importance of discriminating between signal intensity and mere presence of the signal. We are aware of the caveat of targeting enzymes that are regulated on a transcriptional and translational level and hence whose antibody derived signal intensity varies upon different conditions. Therefore, it is of utmost importance to use the most sensitive antibody and imaging setup possible. These measures ensure with high probability that structure identification (neurons) is based not on the signal intensity (i.e. expression level of TH) but solely on the presence or absence of the signal. We believe that using TH antibody and confocal imaging, we achieved the highest sensitivity currently attainable. We tested other antibodies as well, yet we did not find any pan neuronal or sympathetic neuron specific antibody even closely matching the sensitivity of the used anti-tyrosine hydroxylase antibody. As we agree with the reviewer, that this is a very important point we more thoroughly discuss this in the revised manuscript: *"In our hands, of all the antibodies tested, TH was the most sensitive option for staining sympathetic neurons. This is crucial, as axonal outgrowth predominantly affects only the smallest neurons. One major caveat is that TH levels rise during cold exposure, so highly sensitive imaging is essential to avoid confusion from increased neuron detectability caused by higher protein expression. The fact that we did not detect increased neuron density in cold-exposed mice indicates that we achieved a sensitivity level sufficient to detect the smallest axons which are most likely to change during metabolic adaptation. In comparison to light sheet microscopy (Cao et al., 2018; Xie et al., 2022), the use of confocal laser scanning microscopy, as employed in our study and by others (Blaszkiewicz et al., 2019; Willows et al., 2021), allows higher sensitivity and resolution and leads to the capture of finer structures. However, it is thinkable that future, even more sensitive approaches may challenge our current views."*

5) in Lines 208-9 the authors claim adipose morphology is preserved with this approach - this is not demonstrated and is tough to believe. Clearing methods are well known to affect tissue morphology (swelling, distortion) and this is not adequately addressed; even if it is a caveat it would not discount the method and should be honestly discussed. For example, Disco-approaches are known to shrink and harden tissues.

We are grateful for the reviewer's attention on that issue, as addressing it improved the revised manuscript. We tackled adipose tissue morphology determination by measuring adipocyte diameter prior and after the tissue clearing procedure. Any swelling or distortion of the tissue would affect adipocyte morphology and hence diameter. We did not find significant changes in adipocyte size using our methanol free protocol (THF), but we found significant adipocyte shrinkage using the methanol containing protocol (-2.8%) and Adipo-clear (-15.0%) (**Attached Figure 7 and Figure 1H-J** in the revised manuscript). In our clearing approach, similarly to iDISCO we find hardening and added this information to the manuscript. Yet we believe that the tissue-hardening helps to keep the original morphology of the tissue during the delipidation process. We now write in the manuscript: *"All protocols applied increased the stiffness of WAT (not shown). Moreover, we found significant adipocyte shrinkage using our MeOH- (-2.8%) and the Adipo-clear-protocol (-15.0%) but no difference in adipocyte size using our THF- protocol (Fig. 1H-J)."*

© 2025. Published by The Company of Biologists under the terms of the Creative Commons Attribution License (https://creativecommons.org/licenses/by/4.0/).

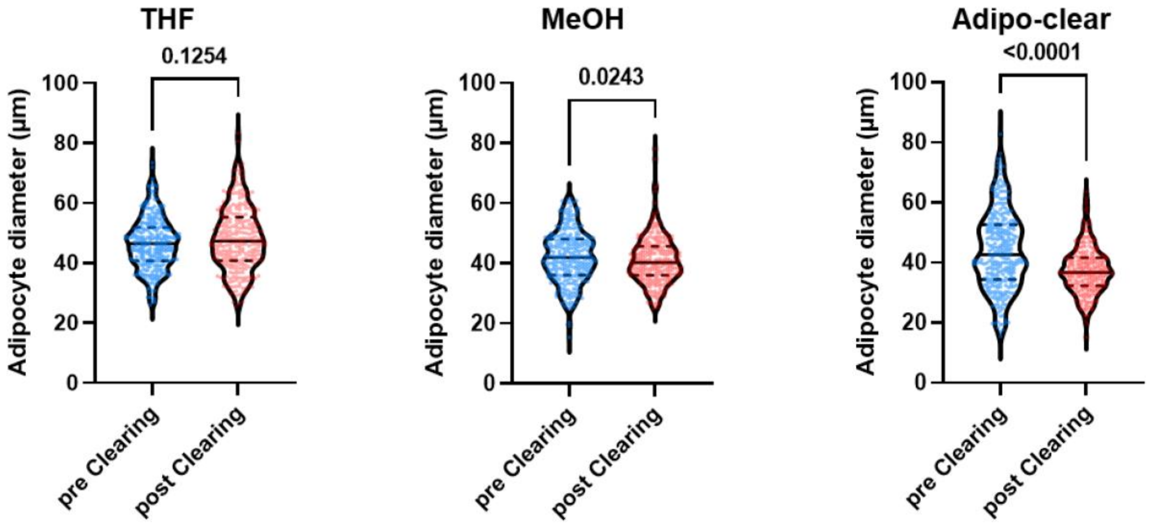

Attached Figure 7: Violin plots with median (solid line) and quartiles (dashed lines) showing adipocyte diameter before and after clearing with the THF-protocol, the MeOH-protocol or Adipo-clear. 300 adipocytes across five randomly selected regions in three biological replicates per group were measured. A Shapiro-Wilk test was performed to test for normal distribution, then a Mann-Whitney U test was performed to analyze the non-normally distributed data.

Changes in response to the autofluorescent layer described in the first draft of the manuscript:

We noticed that the autofluorescent layer described in Supplemental figure 1C,D of the original manuscript showed to be a specific problem that consistently but solely occurred in combination with anti-CD31 antibody and not with any other antibody combination. This fluorescence is likely not unspecific binding alone, since the fluorescence spectrum does not fit the typically expected emission spectrum for the anti-CD31 secondary label. The "non-specific fluorescence", however, was greatly reduced upon intracardially perfusing mice, indicating that there is a connection to remaining erythrocytes or their remnants with the antibody-specific artefact. To address that issue we now discuss that for some antibodies intracardiac perfusion might be necessary to avoid unspecific fluorescent signals: *"While transcardiac perfusion appears essential for clearing blood- and muscle-rich tissues to remove chromophores like haem (Richardson et al., 2021), we found that this step is not generally necessary for adipose tissue. However, certain antibodies may interfere with erythrocytes or erythrocyte remnants and cause unspecific fluorescence signal. In these very specific cases transcardiac perfusion may be warranted."*

<u>Second decision letter</u>

MS ID#: jcs.263438R1

MS Title: Clearing the path for whole-mount labeling and quantification of neuron- and vessel-density in adipose tissue

Authors: Thomas Rauchenwald; Pia Benedikt-Kühnast; Sandra Eder; Gernot F Grabner; Sebastian Forstreiter; Michaela Lang; Roko Sango; Tobias Eisenberg; Thomas Rattei; Arvand Haschemi; Heimo Wolinski; Martina Schweiger

Article Type: Tools and Resources

Dear Dr Schweiger,

© 2025. Published by The Company of Biologists under the terms of the Creative Commons Attribution License (https://creativecommons.org/licenses/by/4.0/).

I am happy to tell you that your manuscript has been accepted for publication in Journal of Cell Science, pending standard publication integrity checks.

Reviewer 1

Advance summary and potential significance to field

Comments for the author

I thank the authors for their detailed responses to my review. The manuscript is greatly improved and I support publication.

© 2025. Published by The Company of Biologists under the terms of the Creative Commons Attribution License (https://creativecommons.org/licenses/by/4.0/).
